# Protein kinase D1 (*Prkd1*) deletion in brown adipose tissue leads to altered myogenic gene expression after cold exposure, while thermogenesis remains intact

**DOI:** 10.14814/phy2.15576

**Published:** 2023-02-17

**Authors:** Mark K. Crowder, Shristi Shrestha, Jean‐Philippe Cartailler, Sheila Collins

**Affiliations:** ^1^ Department of Pharmacology Vanderbilt University School of Medicine Nashville Tennessee USA; ^2^ Creative Data Solutions Shared Resource Vanderbilt University Nashville Tennessee USA; ^3^ Division of Cardiovascular Medicine Vanderbilt University Medical Center Nashville Tennessee USA; ^4^ Department of Molecular Physiology and Biophysics Vanderbilt University, School of Medicine Nashville Tennessee USA

**Keywords:** brown adipocyte progenitors, brown fat/skeletal muscle, mitochondria, β‐adrenergic

## Abstract

Brown adipose tissue (BAT) has in recent times been rediscovered in adult humans, and together with work from preclinical models, has shown to have the potential of providing a variety of positive metabolic benefits. These include lower plasma glucose, improved insulin sensitivity, and reduced susceptibility to obesity and its comorbidities. As such, its continued study could offer insights to therapeutically modulate this tissue to improve metabolic health. It has been reported that adipose‐specific deletion of the gene for protein kinase D1 (*Prkd1*) in mice enhances mitochondrial respiration and improves whole‐body glucose homeostasis. We sought to determine whether these effects were mediated specifically through brown adipocytes using a *Prkd1* brown adipose tissue (BAT) *Ucp1*‐Cre‐specific knockout mouse model, *Prkd1* ^*BKO*^. We unexpectedly observed that upon both cold exposure and β_3_‐AR agonist administration, *Prkd1* loss in BAT did not alter canonical thermogenic gene expression or adipocyte morphology. We took an unbiased approach to assess whether other signaling pathways were affected. RNA from cold‐exposed mice was subjected to RNA‐Seq analysis. These studies revealed that myogenic gene expression is altered in *Prkd1* ^*BKO*^ BAT after both acute and extended cold exposure. Given that brown adipocytes and skeletal myocytes share a common precursor cell lineage expressing *myogenic factor 5* (*Myf5*), these data suggest that loss of *Prkd1* in BAT may alter the biology of mature brown adipocytes and preadipocytes in this depot. The data presented herein clarify the role of *Prkd1* in BAT thermogenesis and present new avenues for the further study of *Prkd1* function in BAT.

## Graphical abstract

The primary goal of these studies was to determine whether loss of PRKD1 in uncoupling protein‐1 (UCP1)‐expressing adipocytes altered the β‐adrenergic receptor (β‐AR)‐stimulated brown adipose tissue (BAT) thermogenesis program since it had been previously reported that adipose‐specific deletion of the gene for *Prkd1* in mice enhanced mitochondrial respiration and improved whole‐body glucose homeostasis. We find that Prkd1^*BKO*^ mice show no differences in brown adipocyte thermogenic gene expression, histology, and morphology compared with their Prkd1^*fl/fl*^ littermates. However, unbiased RNA‐Seq studies showed that upon cold exposure, *Prkd1* loss in BAT significantly altered myogenic gene signatures in the Prkd1^*BKO*^ mice.

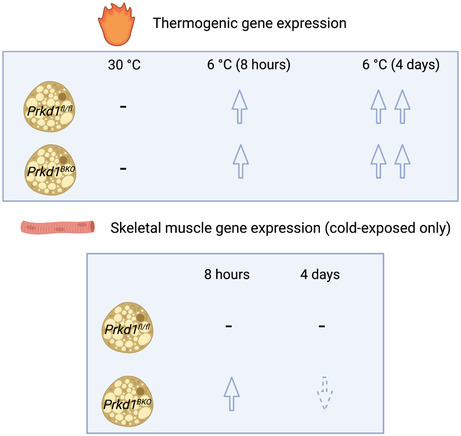

## 1 INTRODUCTION

The study of brown adipose tissue (BAT) has consistently revealed its beneficial metabolic effects both in rodents and humans. The high levels of respiration that occur in BAT provide a mechanism by which it carries out its principal function: thermogenesis or heat production. In fact, the improved insulin sensitivity and reduced percent body fat observed with increased BAT mass or activity are attributed to the high basal respiratory capacity of BAT (Chondronikola et al., 2014; Hamann et al., 1996; Stanford et al., 2013). Research efforts focused on BAT physiology have led to many discoveries from the positive regulation of BAT activity by adrenaline and other hormones to the intracellular signaling effectors that ultimately drive enhanced BAT respiration (Collins, 2022; Collins & Surwit, 2001; Shi & Collins, 2017). Work from our laboratory has shown that p38α MAPK and mechanistic target of rapamycin complex 1 (mTORC1) are key intracellular mediators of β‐adrenergic receptor‐stimulated BAT activity (Cao et al., 2001, 2004; Liu et al., 2016a). However, the additional downstream effectors of these central signaling mediators in β‐adrenergic receptor (β‐AR)‐stimulated BAT activity is unknown. We sought to identify these downstream effectors using phosphoproteomics in cultured brown adipocytes. Proteins with phosphorylation events that were enhanced after stimulation with isoproterenol (a pan β‐AR agonist) and then reduced after rapamycin (an mTORC1 inhibitor) treatment were considered potential substrates of β‐AR‐stimulated mTORC1; insulin +/− rapamycin‐stimulated cells were used to control for canonical mTORC1 activation. These studies showed that protein kinase D1 (PRKD1) was a potential downstream mediator of β‐AR‐stimulated mTORC1 signaling in brown adipocytes.

Work from Loffler et al. (2018) suggested a role for PRKD1 in regulating energy expenditure in mouse adipose tissue. Using a *Prkd1* floxed mouse model crossed with AdipoQ‐Cre mice, they reported that mice lacking *Prkd1* in adipocytes displayed improved insulin sensitivity and glucose tolerance after high‐fat diet feeding. Additionally, they reported that differentiated inguinal adipose stromal vascular cells lacking *Prkd1* had basal increases in uncoupling protein‐1 (*Ucp1*) expression that could be further potentiated by stimulation with the pan β‐AR agonist isoproterenol. A second study (Li et al., 2021) reported that deletion of *Prkd1* in mouse adipocytes had reduced the expression of enzymes in the de novo lipogenesis pathway. However, since they used Fabp4‐Cre (aP2‐Cre) to delete *Prkd1*, and this Cre‐driver has been shown to be expressed in a number of cell types other than adipocytes (Jeffery et al., 2014; Lee et al., 2013; Mullican et al., 2013), results using this model must be treated with caution.

PRKD1 is a member of the Protein Kinase D subfamily of calcium/calmodulin‐dependent protein kinase (CaMK) family of kinases (Rozengurt et al., 2005). Originally named protein kinase Cμ, there are three members of the Protein Kinase D subfamily: PRKD1, 2, and 3. Regulation of catalytic activity and subcellular localization of PRKD1 has been widely studied in cell culture models and more recently, although to a lesser extent, in animal models that have demonstrated the role of PRKD1 in various physiological processes including responses to cardiac remodeling after injury (Fielitz et al., 2008), skeletal muscle endurance (Kim et al., 2008), and insulin secretion (Bergeron et al., 2018; see Renton et al., 2021 for review). Many studies on PRKD1 have been focused on how the enzyme itself is regulated (phosphorylation, kinase activity, etc.; Steinberg, 2012) but there is still much to be understood about the role of PRKD1 in a variety of physiological processes, including in brown/beige adipocytes. In the few papers examining a role for PRKD1 in adipocyte biology (Li et al., 2021; Loffler et al., 2018), important standard maneuvers to study BAT thermogenesis and adipose ‘browning’, such as cold exposure or treatment with a selective β_3_‐AR agonist were not performed. This gap in knowledge, coupled with the relatively high expression of *Prkd1* in mouse iBAT (http://biogps.org/#goto=genereport&id=18760), led us to ask whether loss of *Prkd1* specifically in brown and beige adipocytes (i.e., UCP1‐expressing cells) would modulate β‐AR‐stimulated brown adipose tissue thermogenesis.

Much of the published work in this unique tissue has thus been appropriately focused on efforts to modulate the function of mature brown adipocytes, the parenchymal cell of BAT. However, BAT is composed of numerous cell types including immune cells (macrophages, T cells, etc.), fibroblasts, adipocyte stem cells, and the cells composing its dense vascular and neural networks (endothelial, smooth muscle, and nerve cells among others) (Oguri & Kajimura, 2020; Shinde et al., 2021). While most experiments performed in this study measured phenotypes classically attributed to mature brown adipocytes, RNA‐sequencing studies in cold‐exposed mice revealed *Prkd1*‐dependent changes in myogenic gene expression in BAT. The only cell type in BAT known to possess a myogenic gene signature is the adipocyte precursor, a stem cell (Schulz et al., 2011; Seale et al., 2008; Timmons et al., 2007). While the results of this study show that *Prkd1* deletion in BAT does not modulate phenotypes classically attributed to mature brown adipocytes, our data suggest that mature brown adipocytes lacking *Prkd1* may regulate brown adipocyte precursor cell function in a non cell‐autonomous way.

## 2 MATERIALS AND METHODS

### 2.1 Animal experiments

*Prkd1* ^*fl/fl*^ mice were obtained from Eric Olson (UT Southwestern) and Jens Fielitz (MDC for Molecular Medicine in the Helmholtz Association, Berlin, Germany) and were crossed to mice expressing an uncoupling protein 1 (*Ucp1*)‐driven Cre recombinase (JAX stock no. 024670), resulting in *Prkd1* deletion only in brown and beige adipocytes in these animals (*Prkd1* ^*BKO*^). All mice used for experiments were males between 12 and 14 weeks of age. See Figure S1 for validation of *Prkd1* deletion in the whole iBAT.

#### 2.1.1 Cold exposure

*Prkd1* ^*fl/fl*^ and *Prkd1* ^*BKO*^ mice were housed at thermoneutrality (30°C) in a temperature‐controlled chamber (Powers Scientific) for 2 days, whereupon the temperature was lowered to 6°C for 8 h. This protocol was developed to reduce adrenergic signaling, thus minimizing kinase activation prior to cold exposure (Cao et al., 2004). A control group for each genotype was acclimated at 30°C for 2 days without cold exposure. At the end of the study, the iBAT was dissected and immediately placed in Trizol (ThermoFisher). For the 4‐day cold exposure experiment, mice were housed at thermoneutrality for 2 days followed by 4 days of cold (6°C) exposure. Controls were acclimated at 30°C without cold exposure.

#### 2.1.2 β_3_‐AR agonist (CL316,243) administration

*Prkd1* ^*fl/fl*^ and *Prkd1* ^*BKO*^ mice were administered 0.3 mg/kg BW CL316,243 (Tocris) intraperitoneally once daily for 4 days. On day 5, iBAT and iWAT were dissected and immediately placed in Trizol (ThermoFisher). Similar CL316,243 treatments in mice have been performed in the lab (Ceddia et al., 2021; Liu et al., 2016b).

#### 2.1.3 Body temperature

*Prkd1* ^*fl/fl*^ and *Prkd1* ^*BKO*^ mice were acclimated at thermoneutrality for 2 days followed by 4 days of cold (6°C) exposure. Rectal temperatures were taken every day (including during thermoneutral acclimation) using the PhysiTemp® TH‐5 Thermalert thermometer and RET‐3 rectal probe for mice. Temperature measurements were made between 12 and 2 pm each day.

### 2.2 RNA isolation and quantitative PCR

Total RNA was extracted from adipose tissues using Trizol followed by purification on Qiagen RNA mini‐columns. For qPCR, reverse transcription (High Capacity cDNA reverse transcription kit, ThermoFisher) and cDNA amplification detected by SYBR Green (PowerUp SYBR Green Master Mix, Applied Biosystems) were performed according to manufacturer protocols. qPCR primer sequences are shown in Table 1. qPCR data were analyzed in consultation with the Vanderbilt Biostatistics Clinic using a modified Livak method (Livak & Schmittgen, 2001). *C*_*t*_ values for target genes were normalized to *C*_*t*_ values for 36B4 (reference gene) to obtain a Δ*C*_*t*_ value. Δ*C*_*t*_ values were plotted as relative fold change values. A two‐way analysis of variance (ANOVA) + Tukey's honestly significant difference test were used for statistical analysis. The number of asterisks (*) shown in each graph indicates the level of significance.

**TABLE 1:** qRT‐PCR primers

	Forward (5′–3′)	Reverse (5′–3′)
mPrkd1	AAAATGTGGATATCAGCACAG	ACGATGTTTACCTCCATAAAC
mUcp1	GGCCTCTACGACTCAGTCCA	TAAGCCGGCTGAGATCTTGT
mPgc1α	GAAAGGGCCAAACAGAGAGA	GTAAATCACACGGCGCTCTT
mCidea	GTCTGCAAGCAACCAAAGAT	ATTGAGACAGCCGAGGAAGT
mElovl3	ACTTCGAGACGTTTCAGGACTTA	GACGACCACTATGAGAAATGAGC
mNdufa5 (C1)	GCGGAGCCAGATGTTAAAAA	CCATCCACCATCTGACACTG
mSdhb (CII)	CTGGTGGAACGGAGACAAGT	GTTAAGCCAATGCTCGCTTC
mUqcrb (CIII)	GGGGTGACCCTGAGTATTGA	ATGTAAGGCACCCAGTCCAG
mCox5b (CIV)	CAGAAGGGACTGGACCCATA	ATAACACAGGGGCTCAGTGG
mAtp5k (CV)	CGGTTCAGGTCTCTCCACTC	TGACGCCTCACTTGAGAATG

### 2.3 RNA‐Seq

Another cohort of *Prkd1* ^*fl/fl*^ and *Prkd1* ^*BKO*^ mice were housed at thermoneutrality (30°C) for 2 days +/− 8 h or 4 days cold (6°C) exposure. iBAT RNA was isolated by Trizol (ThermoFisher) and Qiagen RNA extraction kit and sent to Vanderbilt Technologies for Advanced Genomics (VANTAGE) for RNA quality control assessment, library preparation, and next‐generation sequencing. Only high‐integrity (RIN >7) poly‐A‐selected RNA was used as input. Data analysis (including differential gene expression and pathway analyses) was performed by Creative Data Solutions, a Vanderbilt‐shared resource. An Illumina NovaSeq 6000 was used to produce paired‐end, 150‐bp reads yielding 35–45 million reads per sample. Three replicates for each genotype in both thermoneutral and cold exposure states were included. Principal component and distance matrix analyses are shown in Figures S3 and S4, respectively. The mice used in these studies were also used in the respective qPCR experiments, adding to the total number of replicates for the latter experiments. The RNA‐Seq data that support this study are available in the ArrayExpress repository for both raw and processed data via accession ID E‐MTAB‐12170.

### 2.4 Bioinformatics analysis of RNA‐seq

Paired‐end raw fastq files were assessed for quality by FASTQC (https://www.bioinformatics.babraham.ac.uk/projects/fastqc/) and TrimGalore (https://www.bioinformatics.babraham.ac.uk/projects/trim_galore/), respectively. Reads were aligned to the reference mouse genome mm10 (GRCm38) using The Spliced Transcripts Alignment to a Reference (STAR) version 2.6 (Dobin et al., 2013). Approximately 70% of the raw reads were uniquely mapped to the reference genome. Raw read counts were obtained from STAR followed by pairwise differential gene expression analysis performed using DESeq2 (Love et al., 2014). Genes with an adjusted *p* value of <0.05 were considered significant. Gene Ontology analysis and visuals were performed using the clusterProfiler R package (Yu et al., 2012). Metascape network visualizations of statistically enriched GO terms were performed as previously described (Zhou et al., 2019).

### 2.5 Histology

Adipose tissues were fixed in 10% buffered formalin, embedded in paraffin, and sectioned (5‐μm thickness). Slides were subjected to either UCP1 immunohistochemistry (IHC) or hematoxylin and eosin (H&E) staining. Images were captured using an Aperio AT2 digital slide scanner (20× magnification).

## 3 RESULTS

**FIGURE 1:**
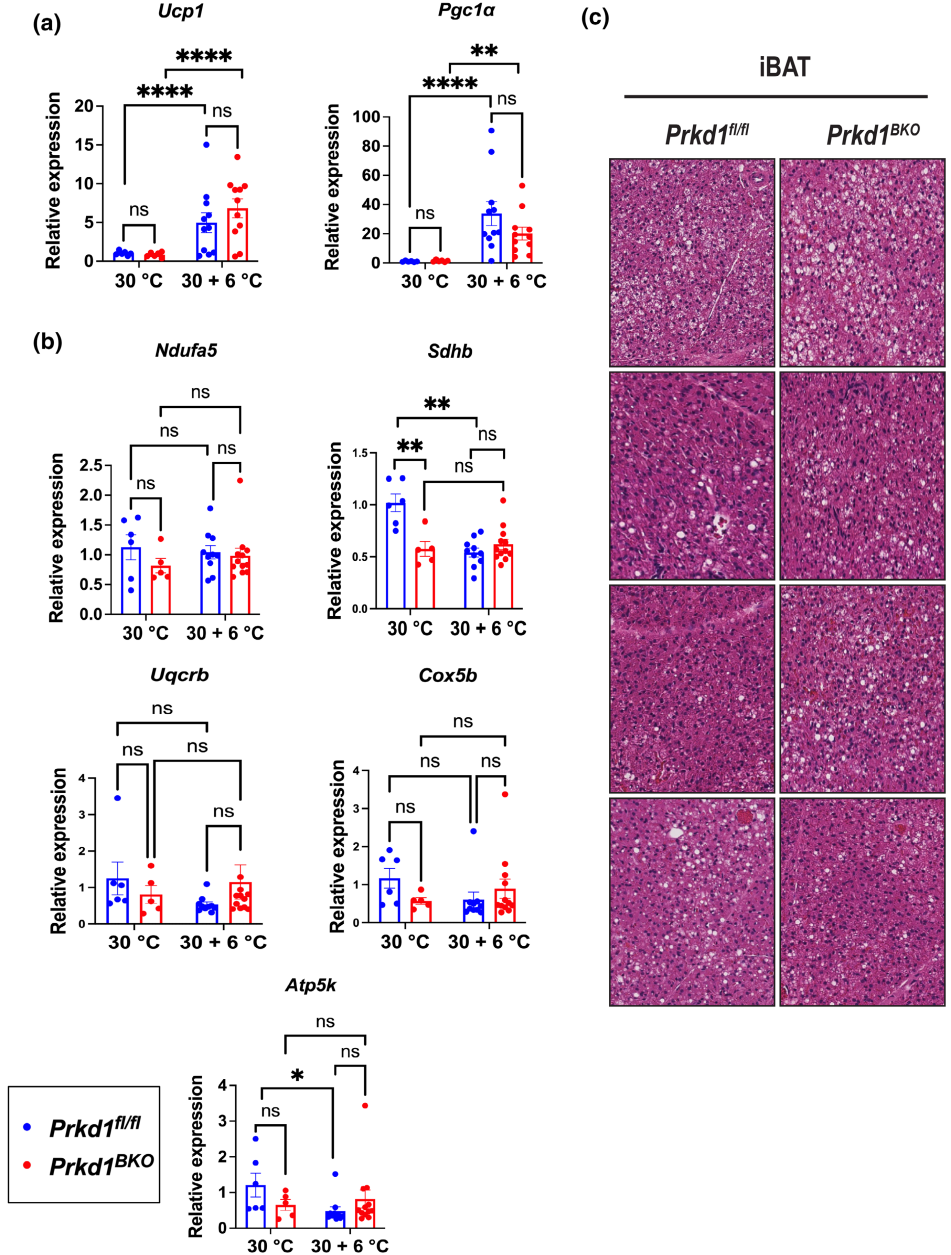
Eight‐hour cold exposure reveals similar thermogenic gene induction in iBAT between *Prkd1* ^*fl/fl*^ and *Prkd1* ^*BKO*^ mice. *Prkd1* ^*fl/fl*^ and *Prkd1* ^*BKO*^ mice were acclimated at 30°C (thermoneutrality) for 2 days with or without an additional 8 h at 6°C (cold). (a) *Ucp1* and *Pgc1α* expression in iBAT. (b) Expression of *Ndufa5*, *Sdhb*, *Uqcrb*, *Cox5b*, and *Atp5k* representing, in order, subunits of mitochondrial complexes I–V in iBAT. *n* = 6–11 mice. Data are presented as mean ± SEM (two‐way ANOVA with Tukey's honestly significant difference test). (c) *Prkd1* ^*fl/fl*^ and *Prkd1* ^*BKO*^ mice were housed at 30°C for 2 days followed by 8 h at 6°C. iBAT was dissected for fixation and paraffin embedding followed by hematoxylin and eosin (H & E) staining. Adipocyte morphology was assessed by a trained pathologist at the Translational Pathology core, Vanderbilt University Medical Center. *n* = 5 mice per genotype.

The primary goal of these studies was to determine whether loss of PRKD1 in UCP1‐expressing adipocytes altered β‐AR‐stimulated BAT thermogenesis. Since mice are typically housed at 22–25°C, which is moderate thermal stress for a mouse, we chose to first acclimate *Prkd1* ^*fl/fl*^ and *Prkd1* ^*BKO*^ mice at thermoneutrality (30°C) for 2 days to minimize catecholaminergic tone. In the first study, this was followed by 8 h at 6°C. A control group of both genotypes was housed at 30°C only. As shown in Figure 1a, RT‐PCR analysis showed that cold exposure led to similar increases in the expression of *Ucp1* and PPAR‐gamma coactivator‐1α (*Pgc1α*), key genes involved in the thermogenic response in adipose tissue, in iBAT of both *Prkd1* ^*fl/fl*^ and *Prkd1* ^*BKO*^ mice. Also, the expression of mitochondrial complex genes was similar between genotypes after cold exposure (Figure 1b), suggesting that the loss of PRKD1 in brown adipocytes does not affect the acute thermogenic response to cold. One notable difference we observed here was that succinate dehydrogenase complex iron sulfur subunit B (*Sdhb*) expression was significantly downregulated in *Prkd1* ^*BKO*^ mice relative to *Prkd1* ^*fl/fl*^ mice at 30°C only. Also, expression of *Sdhb* was reduced in cold‐exposed *Prkd1* ^*fl/fl*^, but not *Prkd1* ^*BKO*^, mice compared with *Prkd1* ^*fl/fl*^ mice at 30°C only. In inguinal WAT (iWAT), the expression of *Ucp1* and *Pgc1α* was not increased after cold exposure in either genotype (data not shown), likely due to the short duration of cold exposure. H&E staining of iBAT from mice either housed at thermoneutrality or after 8‐h cold exposure revealed no PRKD1‐dependent differences in adipocyte morphology (Figure 1c). Taken together with the gene expression analysis, these data suggest that *PRKD1* is not a key regulator of the acute thermogenic response in iBAT.

**FIGURE 2:**
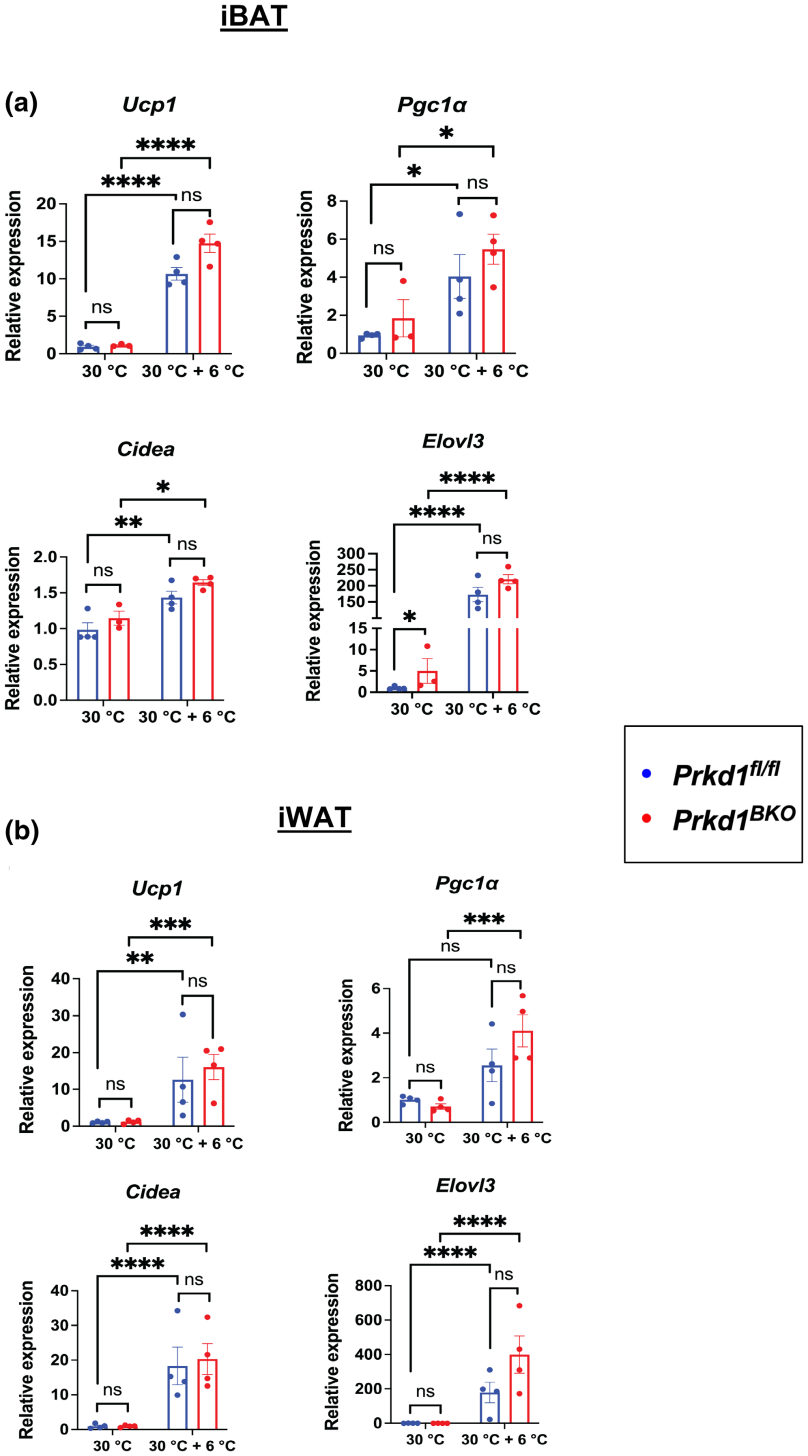
Four‐day cold‐exposed *Prkd1* ^*fl/fl*^ and *Prkd1* ^*BKO*^ mice have no significant differences in thermogenic gene induction in either iBAT or iWAT. *Prkd1* ^*fl/fl*^ and *Prkd1* ^*BKO*^ mice were acclimated at 30°C (thermoneutrality) for 2 days with or without an additional 4 days at 6°C (cold). (a) *Ucp1, Pgc1α*, *Cidea*, and *Elovl3* expression in iBAT. (b) *Ucp1*, *Pgc1α*, *Cidea*, and *Elovl3* expression in iWAT. *n* = 4 mice/group. Data are presented as mean ± SEM (two‐way ANOVA with Tukey's honestly significant difference test).

**FIGURE 3:**
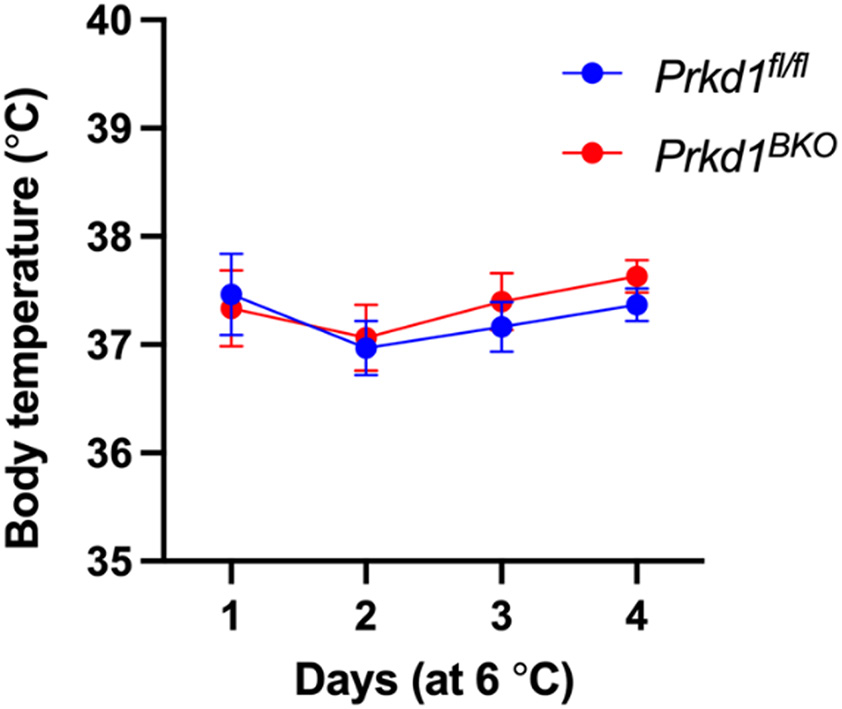
Core body temperature of *Prkd1* ^*fl/fl*^ and *Prkd1* ^*BKO*^ mice during the 4‐day cold exposure. *Prkd1* ^*fl/fl*^ and *Prkd1* ^*BKO*^ mice were acclimated at 30°C for 2 days followed by an additional 4 days at 6°C (cold). Core body temperature was recorded each day as detailed in Methods. *n* = 4 mice/group.

**FIGURE 4:**
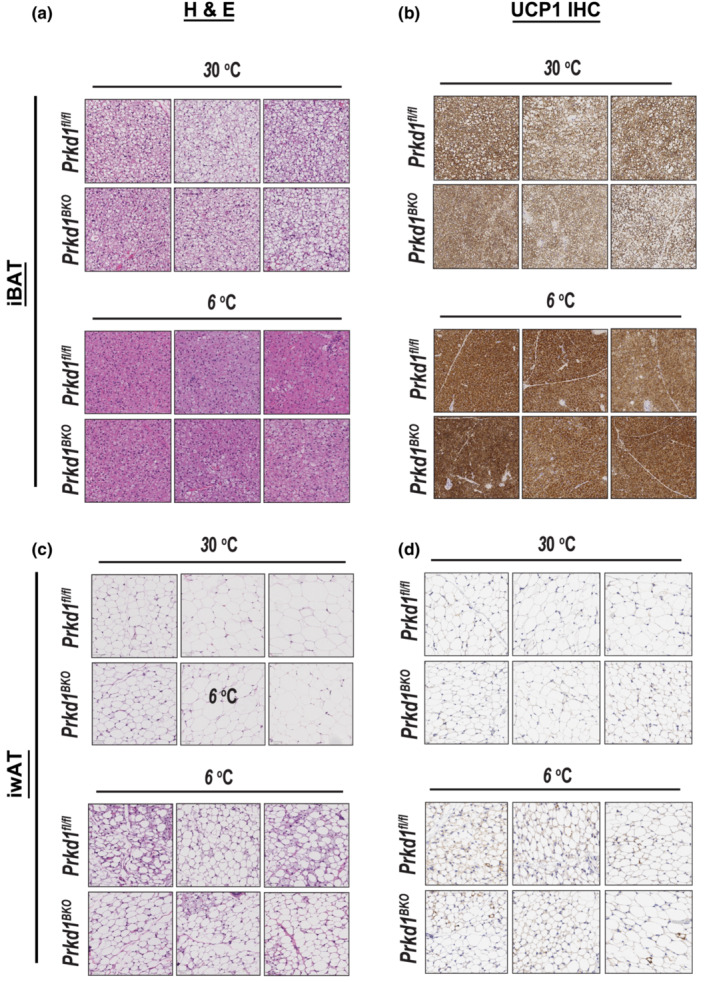
H & E staining and UCP1 immunohistochemistry of iBAT and iWAT after 4‐day cold exposure. *Prkd1* ^*fl/fl*^ and *Prkd1* ^*BKO*^ mice were housed at 30°C for 2 days +/− 4 days at 6°C. iBAT and iWAT were dissected for fixation and paraffin embedding followed by hematoxylin and eosin (H & E) staining and UCP1 IHC. (a) iBAT H&E staining, (b) iBAT UCP1 IHC, (c) iWAT H & E, (d) iWAT UCP1 IHC. *n* = 3 mice per group

We next performed a longer 4‐day cold exposure in *Prkd1* ^*fl/fl*^ and *Prkd1* ^*BKO*^ mice, since more chronic stimulation will further promote brown and beige fat gene expression and thermogenesis. Similar to the results from the 8‐h cold exposure when comparing genotypes, we did not observe PRKD1‐dependent changes in thermogenic gene induction after 4 days at 6°C in either iBAT (Figure 2a) or iWAT (Figure 2b), nor was there any difference in core body temperature between genotypes (Figure 3). In addition, both H&E staining and UCP1 IHC for iBAT were similar between *Prkd1* ^*fl/fl*^ and *Prkd1* ^*BKO*^ mice (Figure 4a,b). In the iWAT, while we observed for the most part the expected increases in gene expression in response to cold, *Pgc1α* expression in the *Prkd1* ^*fl/fl*^ mice did not reach significance (Figure 2b), perhaps due to the variation observed between mice. The data do show, however, that *Pgc1α* expression was upregulated in *Prkd1* ^*BKO*^ mice after cold exposure but not *Prkd1* ^*fl/fl*^ mice, which may indicate a more nuanced role for PRKD1 in the regulation of thermogenic gene expression. Mitochondrial complex gene expression was not assessed at this time point because we did not anticipate changes in their mRNA levels and histological changes indicative of adipose “browning” (Figure 4c,d) after cold exposure, were not different between genotypes.

**FIGURE 5:**
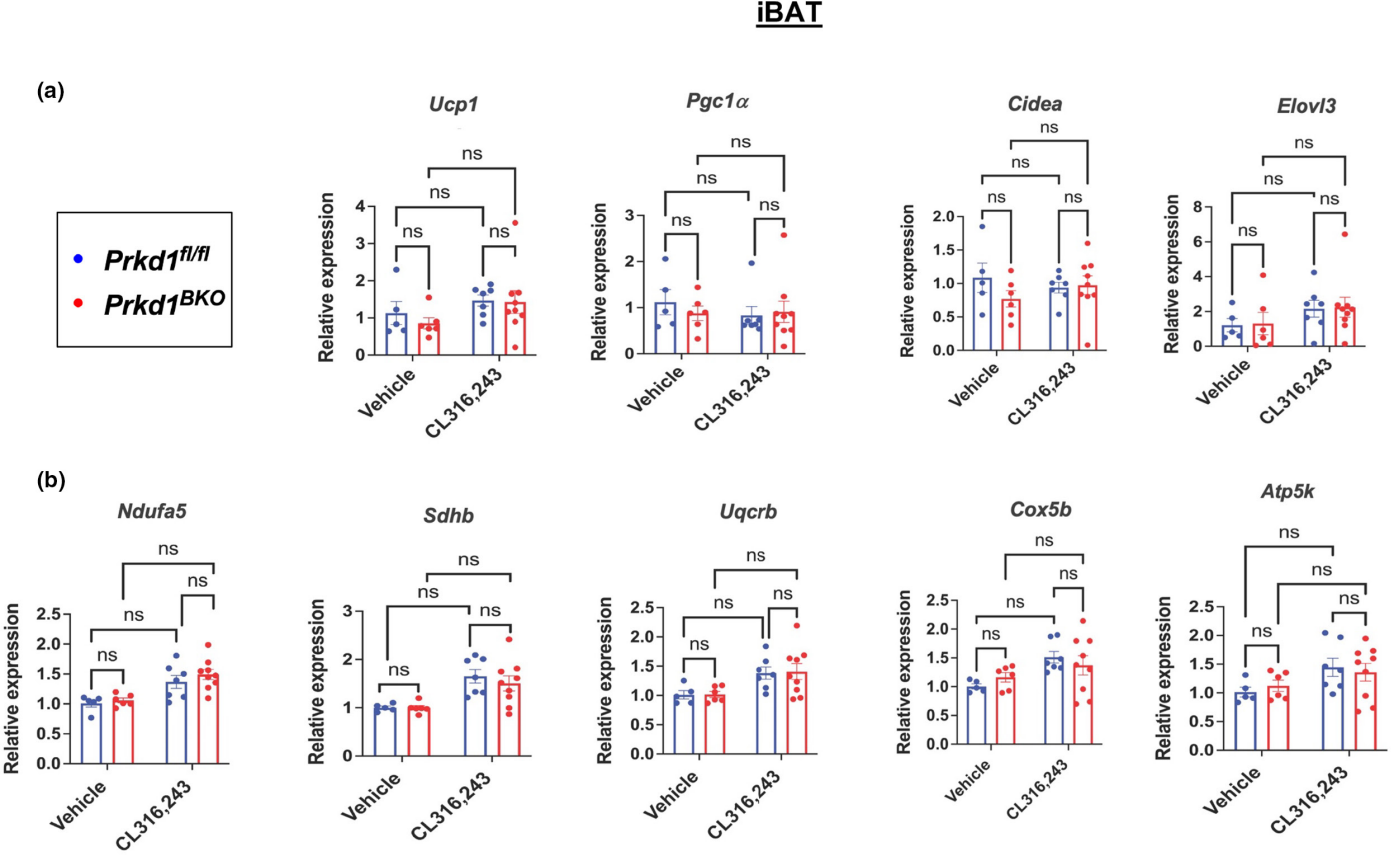
Loss of *Prkd1* in iBAT does not alter β_3_‐adrenergic receptor agonist‐stimulated thermogenic gene expression. *Prkd1* ^*fl/fl*^ and *Prkd1* ^*BKO*^ mice were intraperitoneally injected with 0.3 mg/kg CL316,243 (CL) once daily for 4 days. iBAT and iWAT were harvested on day 5 for RNA isolation and qRT‐PCR. (a) Expression of *Ucp1, Pgc1α*, *Cidea*, and *Elovl3* in iBAT. (b) Expression of *Ndufa5*, *Sdhb*, *Uqcrb*, *Cox5b*, and *Atp5k* representing, in order, subunits of mitochondrial complexes I–V in iBAT.

**FIGURE 6:**
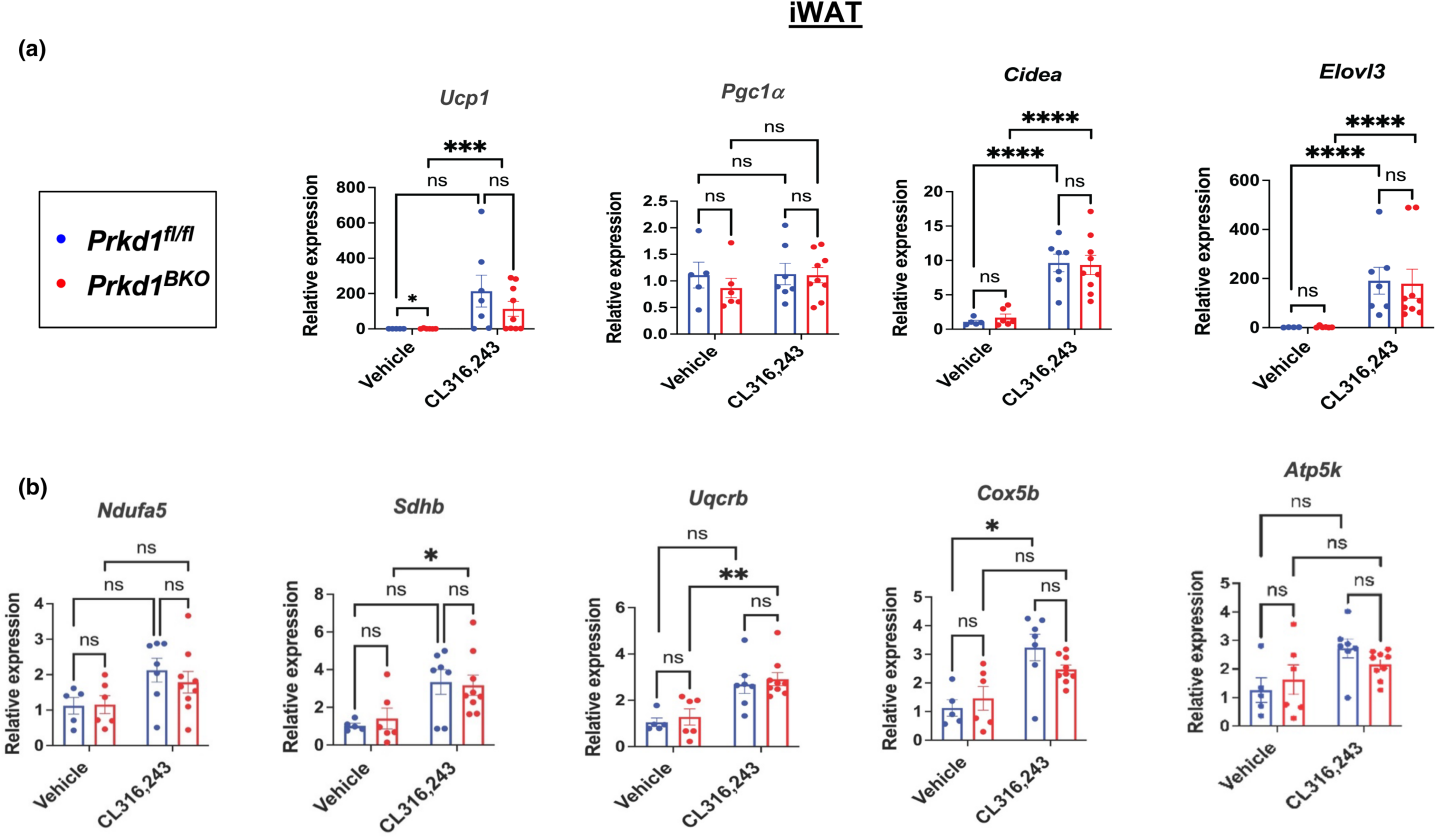
β_3_AR agonist‐stimulated thermogenic gene expression in iWAT of *Prkd1* ^*fl/fl*^ and *Prkd1* ^*BKO*^ mice. (a) Expression of *Ucp1*, *Pgc1α*, *Cidea*, and *Elovl3* in iWAT. (b) Expression of *Ndufa5*, *Sdhb*, *Uqcrb*, *Cox5b*, and *Atp5k* representing, in order, subunits of mitochondrial complexes I–V in iWAT. *n* = 5–9 mice. Data are presented as mean ± SEM (two‐way ANOVA with Tukey's honestly significant difference test).

As a companion experiment to the cold exposure, we took a pharmacological approach using the β_3_‐AR agonist, CL316,243 (CL) to assess the effects of *Prkd1* loss on thermogenic gene induction in iBAT and iWAT. In iBAT, there was no significant increase in thermogenic gene expression (*Ucp1*, *Pgc1α*, *Cidea*, and *Elovl3*) (Figure 5a), nor was mitochondrial gene expression altered in iBAT between genotypes (Figure 5b). We attribute this result to the very high baseline expression of these genes in iBAT since BAT is densely innervated and tonically stimulated by endogenous NE. Furthermore, our hypothesis was that *Prkd1* loss in iBAT would enhance thermogenic gene expression. Assuming an endogenous maximum threshold for the expression/induction of these genes, we used a submaximal dose of CL to produce responses in the dynamic range of the dose–response curve to observe *Prkd1*‐dependent differences that may be obscured at higher doses of CL. However, in iWAT, expression of *Ucp1* was higher in vehicle‐treated *Prkd1* ^*BKO*^ mice relative to vehicle‐treated *Prkd1* ^*fl/fl*^ mice. *Ucp1*, *Sdhb*, and ubiquinol‐cytochrome c reductase binding protein (*Uqcrb*) expression were significantly increased in CL‐treated *Prkd1* ^*BKO*^, but not *Prkd1* ^*fl/fl*^ mice. Cytochrome c oxidase subunit 5B (*Cox5b*) expression was enhanced in *Prkd1* ^*fl/fl*^ mice, but not *Prkd1* ^*BKO*^ mice after CL administration. Overall, thermogenic gene expression (Figure 6a) and some mitochondrial complex genes (Figure 6b) were robustly induced by CL in both genotypes (Figure 6a), but *Prkd1* deficiency did not alter the induction of these genes. These data are consistent with our observations from the acute and 4‐day cold exposure studies, strongly suggesting that *Prkd1* is not a significant regulator of β‐AR‐stimulated thermogenic gene expression in UCP1‐expressing adipocytes. Nevertheless, since in iWAT the expression of Cre recombinase only occurs once endogenous *Ucp1* is induced, we did not observe deletion of *Prkd1* in iWAT in our experimental paradigm. It is possible that a longer period of cold or CL treatment may be needed to see changes in iWAT.

**FIGURE 7:**
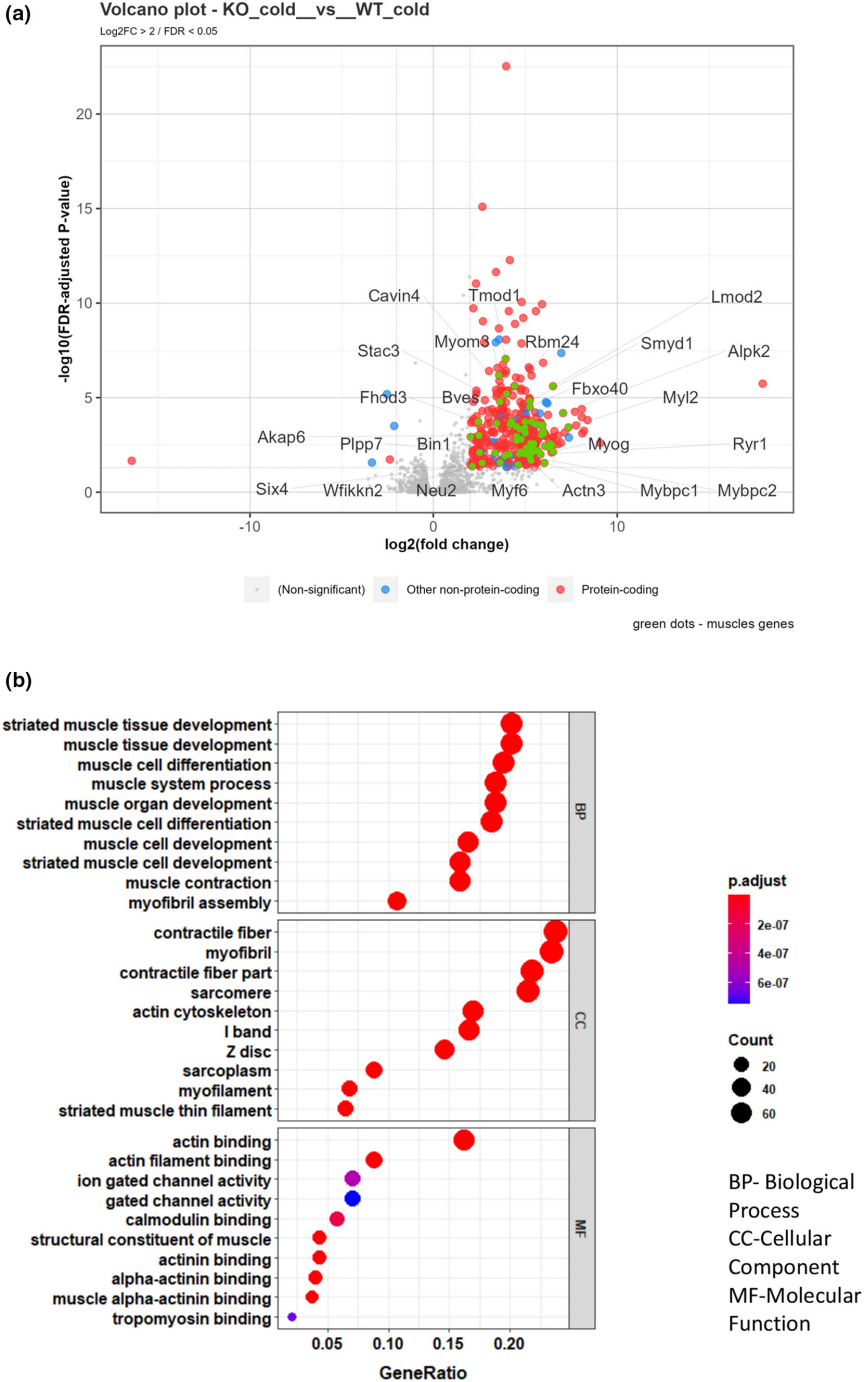
Gene ontology (GO) analysis of iBAT RNAs from *Prkd1* ^*fl/fl*^ and *Prkd1* ^*BKO*^ mice after 8‐h cold exposure. GO plots show biological processes (Muller et al., 2013), cellular components (CC), and molecular functions (MF) changed between the two groups being compared. The GeneRatio indicates the percentage of total differentially expressed genes (DEGs) in each GO term. (a) Volcano plot of DEGs between both genotypes after cold exposure. (b) GO terms for DEGs

Since based on prior literature (Loffler et al., 2018), we provisionally expected to see heightened thermogenic gene expression in *Prkd1* ^*BKO*^ mice, we next performed RNA‐Seq to assess whether other transcriptional changes resulted from *Prkd1* deficiency in iBAT, first using the 8‐h cold exposure paradigm. For both genotypes, we observed comparable increases in the expression of key thermogenic genes (e.g., *Ucp1*, *Pgc1α*, *Dio2*, *Cidea*) in response to the 8‐h cold relative to thermoneutrality (see Figure S2). Thus, as in Figure 1, there were no differences in cold‐induced thermogenic gene induction between genotypes. Instead, what we did observe was a significantly increased myogenic gene signature in the *Prkd1* ^*BKO*^ versus *Prkd1* ^*fl/fl*^ mice after cold exposure (Figure 7). However, there were no differences in this myogenic expression profile between genotypes at the thermoneutral temperature. For a more complete view of the genes and gene families that were changed in this experiment, please see Figure S5 and data source files. This myogenic signature is interesting given that brown adipocytes and skeletal myocytes arise from a common progenitor that expresses Myf5 (Seale et al., 2008; Timmons et al., 2007). The transcriptional regulator PRDM16 has been shown to drive the brown adipocyte differentiation pathway versus skeletal muscle (Harms et al., 2014; Seale et al., 2008). In our data set, there were no differences in the levels of *Prdm16* between *Prkd1* ^*fl/fl*^ and *Prkd1* ^*BKO*^ under any condition. Moreover, since we used bulk RNA‐Seq, these data cannot inform us in what cell type(s) these transcript changes are occurring.

**FIGURE 8:**
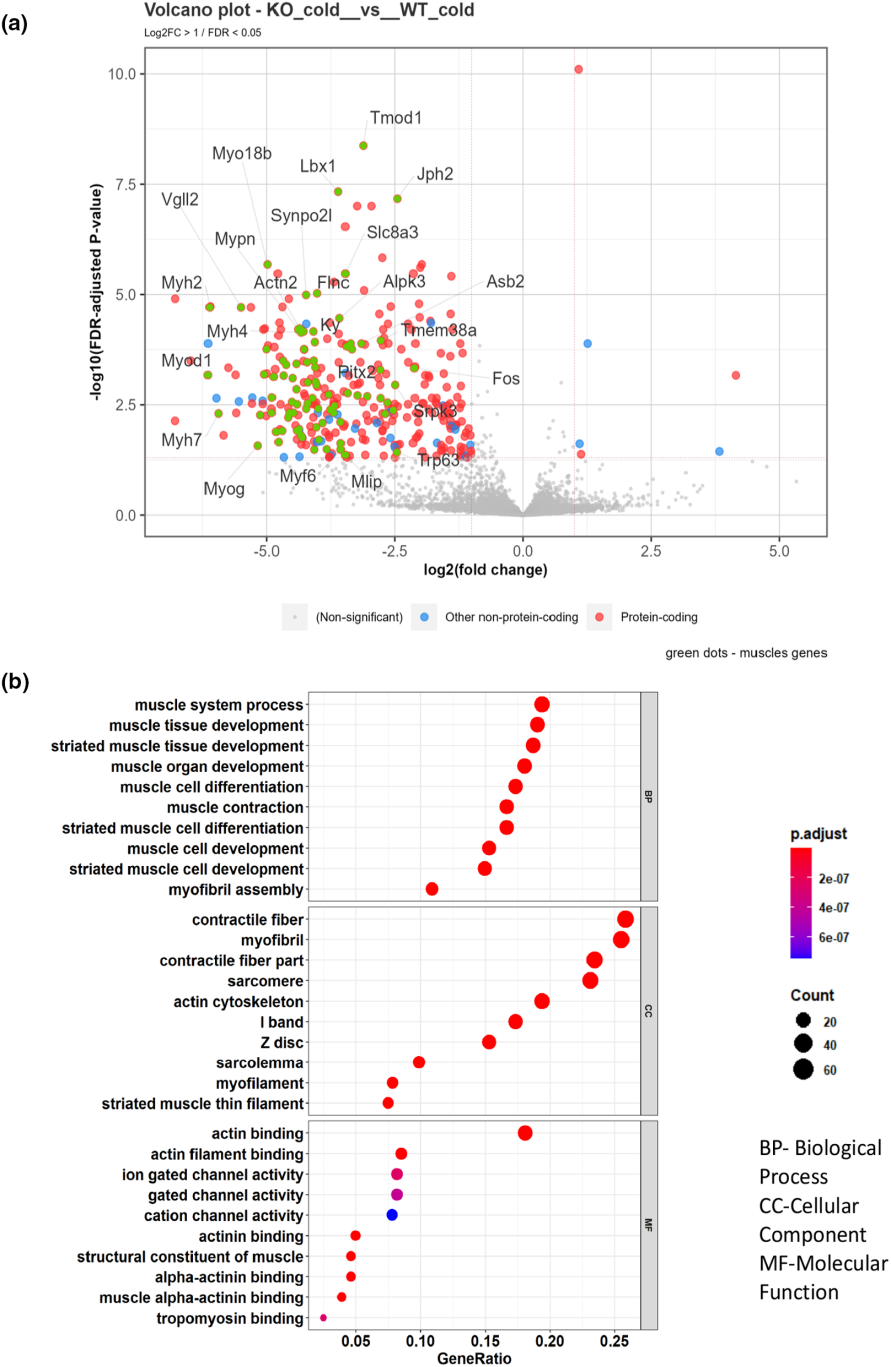
Gene ontology (GO) analysis of iBAT RNAs from *Prkd1* ^*fl/fl*^ and *Prkd1* ^*BKO*^ mice after 4‐day cold exposure. GO plots show biological processes (Muller et al., 2013), cellular components (CC), and molecular functions (MF) changed between the two groups being compared. The GeneRatio indicates the percentage of total differentially expressed genes (DEGs) in each GO term. (a) Volcano plot of DEGs between both genotypes after cold exposure. (b) GO terms for DEGs

Since the data from 8‐h cold exposure provide a snapshot of what may be occurring during this acute time frame, we next employed the longer 4‐day cold exposure paradigm to determine whether other changes may be occurring during the sustained thermogenic stimulus when nonshivering thermogenesis is further established. In both genotypes, we observed equally robust increases in the expression of the canonical genes involved in nonshivering thermogenesis after cold exposure compared with their thermoneutral controls (see Figure S2). These results again independently support the data in Figure 2. Based on our 8‐h cold exposure data, we speculated that perhaps the myogenic gene signature in the iBAT of the *Prkd1* ^*BKO*^ would persist and perhaps be amplified. However, as shown in Figure 8, compared with *Prkd1* ^*fl/fl*^ mice, the *Prkd1* ^*BKO*^ mice in fact displayed a suppressed myogenic gene signature after the 4‐day cold exposure, suggesting that *Prkd1* loss in iBAT has different effects that are dependent on the length of cold exposure. See Figure S6 and ArrayExpress data accession ID E‐MTAB‐12170 for a broader view of the genes and gene families that were changed in this experiment. Another interesting finding from the RNA‐Seq study (8‐h in particular) is that *Prkd1*‐deficient iBAT has modest but significantly reduced lipogenic gene expression after 2‐day acclimation at thermoneutrality.

## 4 DISCUSSION AND CONCLUSIONS

While many similarities exist between mouse and human BAT, there are certain distinctions. For example, in mice, the primary bona fide depot is located between the scapulae (i.e., interscapular BAT) and transplantation of additional BAT into recipient mice resulted in improved glucose metabolism (Stanford et al., 2013). However, in humans, it is only since 2009 that the existence of brown fat was unequivocally demonstrated (Cypess et al., 2009; Saito et al., 2009; van Marken Lichtenbelt et al., 2009; Virtanen et al., 2009), and compared with rodent studies is relatively new. In humans, BAT exists in discretely distributed depots along the neck and spine (Cypess et al., 2009; Kiefer, 2017; Leitner et al., 2017; Saito et al., 2009; van Marken Lichtenbelt et al., 2009; Virtanen et al., 2009) but is still debated as to whether there are brown adipocytes as in rodent iBAT or more of an inducible “beige” type adipocyte (Kiefer, 2017). Another important distinction is that the amount of β_3_AR in rodent adipose tissue is very high versus in humans where it is much lower (Collins & Surwit, 2001). Also, the lack of β_3_‐AR agonist efficacy in human clinical trials, largely due to their pharmacodynamic properties (Danforth & Himms‐Hagen, 1997; Himms‐Hagen et al., 1996) and off‐target effects such as altered cardiovascular function (Wheeldon et al., 1994), suggest that the β_3_‐AR is differentially expressed and/or regulated in humans versus mice (Bloom et al., 1992; Pietri‐Rouxel & Strosberg, 1995; Weyer et al., 1999). Thus, while the mechanistic findings in adipocyte biology are overall comparable between rodents and humans, the magnitude of responses and their penetrance between models should be considered with these differences in mind. Our initial hypothesis in these studies, which was based upon prior literature showing that loss of *Prkd1* in adipose tissue enhanced energy expenditure (Loffler et al., 2018), was that *Prkd1* loss in iBAT would similarly enhance thermogenesis. However, the data presented here show essentially no difference in thermogenic gene expression, histological features, or body temperature between *Prkd1* ^*fl/fl*^ and *Prkd1* ^*BKO*^ mice after either cold exposure or β_3_‐AR agonist administration. Despite findings from Loffler et al. (2018) that loss of *Prkd1* in adipose tissue (both white and brown) improved insulin sensitivity and glucose tolerance as well as potentiated isoproterenol stimulated *Ucp1* expression in cultured adipocytes, our data show that *Prkd1* is not a regulator of iBAT thermogenesis. One potential explanation for this discrepancy is that our animal model (*Prkd1* ^*BKO*^) only deleted *Prkd1* in *Ucp1*‐expressing adipocytes, whereas the model used by Löffler et al. (2018) resulted in *Prkd1* deficiency in all adipose tissue depots. Importantly, Loffler et al. did not examine BAT function in their study. Thus, the difference in model systems may explain why we failed to observe *Prkd1*‐dependent differences in thermogenesis.

Loss of *Prkd1* in BAT did alter myogenic gene expression after both 8 h and 4 days of cold exposure. The 8‐h cold‐exposed *Prkd1* ^*BKO*^ mice had elevated myogenic gene expression relative to 8‐h cold‐exposed *Prkd1* ^*fl/fl*^ mice, while after 4 days of cold exposure, the trend tended to be reversed. Timmons and Seale showed that myogenic gene expression in BAT arises from early adipocyte progenitor cells before their commitment to the adipocyte lineage (Seale et al., 2008; Timmons et al., 2007). Additionally, Seale and colleagues demonstrated that this myogenic signature was inhibited by EBF2 (Rajakumari et al., 2013) and PRDM16 (Seale et al., 2008), two transcription factors that promote brown and beige adipogenesis, allowing adipocyte progenitors to differentiate into mature brown and beige adipocytes. Other than this critical finding, there are no data to explain the expression of a myogenic signature in BAT.

Thus, we hypothesize that during acute (8‐h) cold exposure, loss of *Prkd1* promotes a transcriptional response in BAT that elevates myogenic gene expression, which could be generated by an increase in the number or transcriptional activity of early adipocyte progenitors. After 4 days in the cold, the cold‐exposed *Prkd1* ^*BKO*^ mice have reduced myogenic gene expression relative to *Prkd1* ^*fl/fl*^ cold‐exposed mice. When comparing these changes in myogenic gene expression between the 8‐h and 4‐day cold exposure studies, one reasonable hypothesis is that at the 4‐day time point, a factor (i.e., enzyme, receptor, etc.) compensating for the loss of *Prkd1* in mature brown adipocytes has suppressed the myogenic gene expression. Another possibility is that the differences in myogenic gene expression between 8 h and 4‐days cold exposure could be due to enhanced differentiation of progenitors in the *Prkd1* ^*fl/fl*^ mice in the chronic cold. However, additional in vivo and in vitro experiments are needed to test these hypotheses to confirm both the cell type(s) of origin for the observed myogenic gene signature and its functional relevance in BAT.

## FUNDING INFORMATION

This work was supported by National Institutes of Health grants R01 DK116625 (SC) and NIH R01 DK116625‐01S1 (MKC).

## ETHICAL STATEMENT

All animal experiments were prospectively reviewed and approved by the Institutional Animal Care and Use Committee of the Vanderbilt University Medical Center.

## Supporting information


Figure S1:
**Figure S2**:
**Figure S3**:
**Figure S4**:
**Figure S5**:
**Figure S6**: Click here for additional data file.
