# Supplementary material for: Protein kinase D1 (Prkd1) deletion in brown adipose tissue leads to altered myogenic gene expression after cold exposure, while thermogenesis remains intact
Source: Physiol Rep. 2023 Feb 17;11(4):e15576. doi: 10.14814/phy2.15576 (PMC9937785; doi:10.14814/phy2.15576)
Supplement: Supplementary file 1 — Figure S1: Figure S2: Figure S3: Figure S4: Figure S5: Figure S6: [file PHY2-11-e15576-s001.pdf]

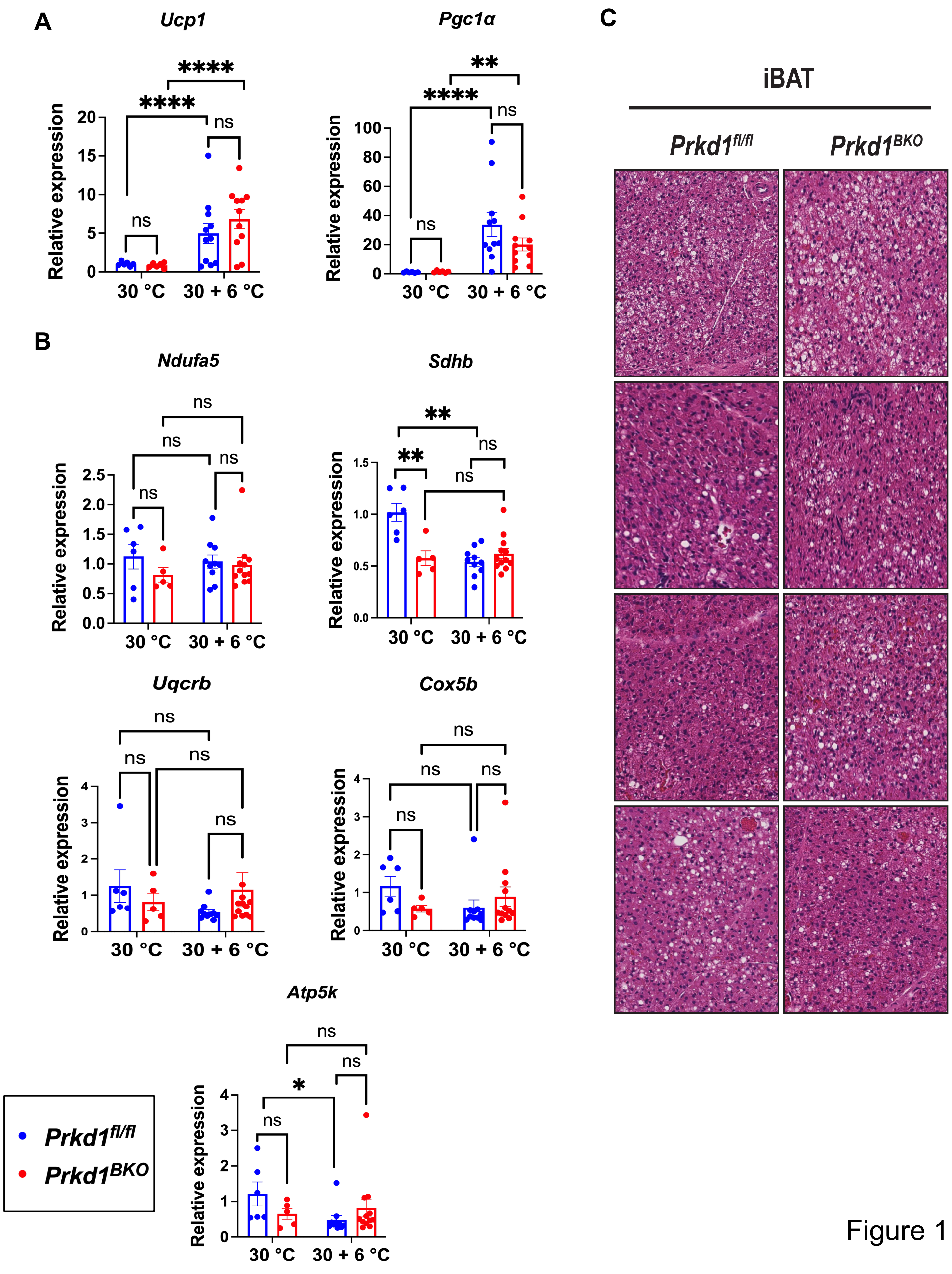

A
Ucp1
Pgc1α
Relative expression
30 °C
30 + 6 °C
ns
****
**
B
Ndufa5
Sdhb
Uqcrb
Cox5b
Atp5k
*
C
iBAT
Prkd1fl/fl
Prkd1BKO

Figure 1

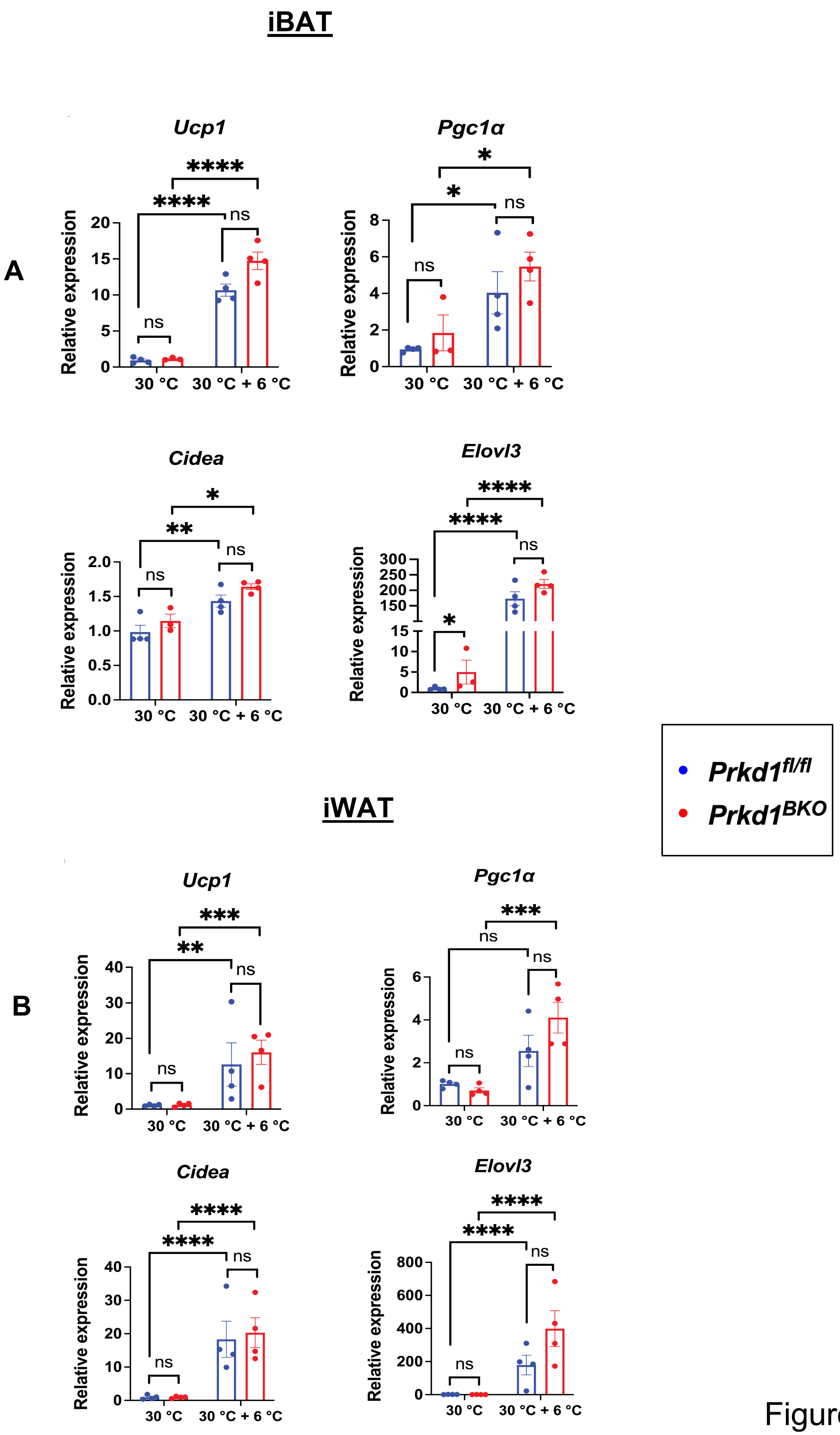

iBAT
A
Ucp1
Relative expression
20
15
10
5
0
****
****
ns
ns
30 °C
30 °C + 6 °C
Pgc1α
Relative expression
8
6
4
2
0
*
*
ns
ns
30 °C
30 °C + 6 °C
Cidea
Relative expression
2.0
1.5
1.0
0.5
0.0
*
**
ns
ns
30 °C
30 °C + 6 °C
Elovl3
Relative expression
300
250
200
150
15
10
5
0
****
****
ns
*
30 °C
30 °C + 6 °C
Prkd1fl/fl
Prkd1BKO
iWAT
B
Ucp1
Relative expression
40
30
20
10
0
***
**
ns
ns
30 °C
30 °C + 6 °C
Pgc1α
Relative expression
6
4
2
0
***
ns
ns
ns
30 °C
30 °C + 6 °C
Cidea
Relative expression
40
30
20
10
0
****
****
ns
ns
30 °C
30 °C + 6 °C
Elovl3
Relative expression
800
600
400
200
0
****
****
ns
ns
30 °C
30 °C + 6 °C

Figure 2

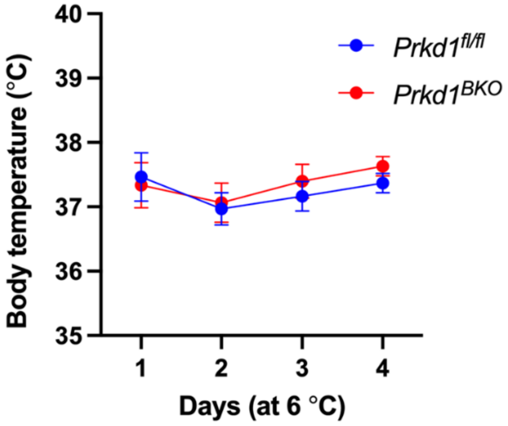

40
39
38
37
36
35
Body temperature (°C)
Prkd1fl/fl
Prkd1BKO
1
2
3
4
Days (at 6 °C)

Figure 3

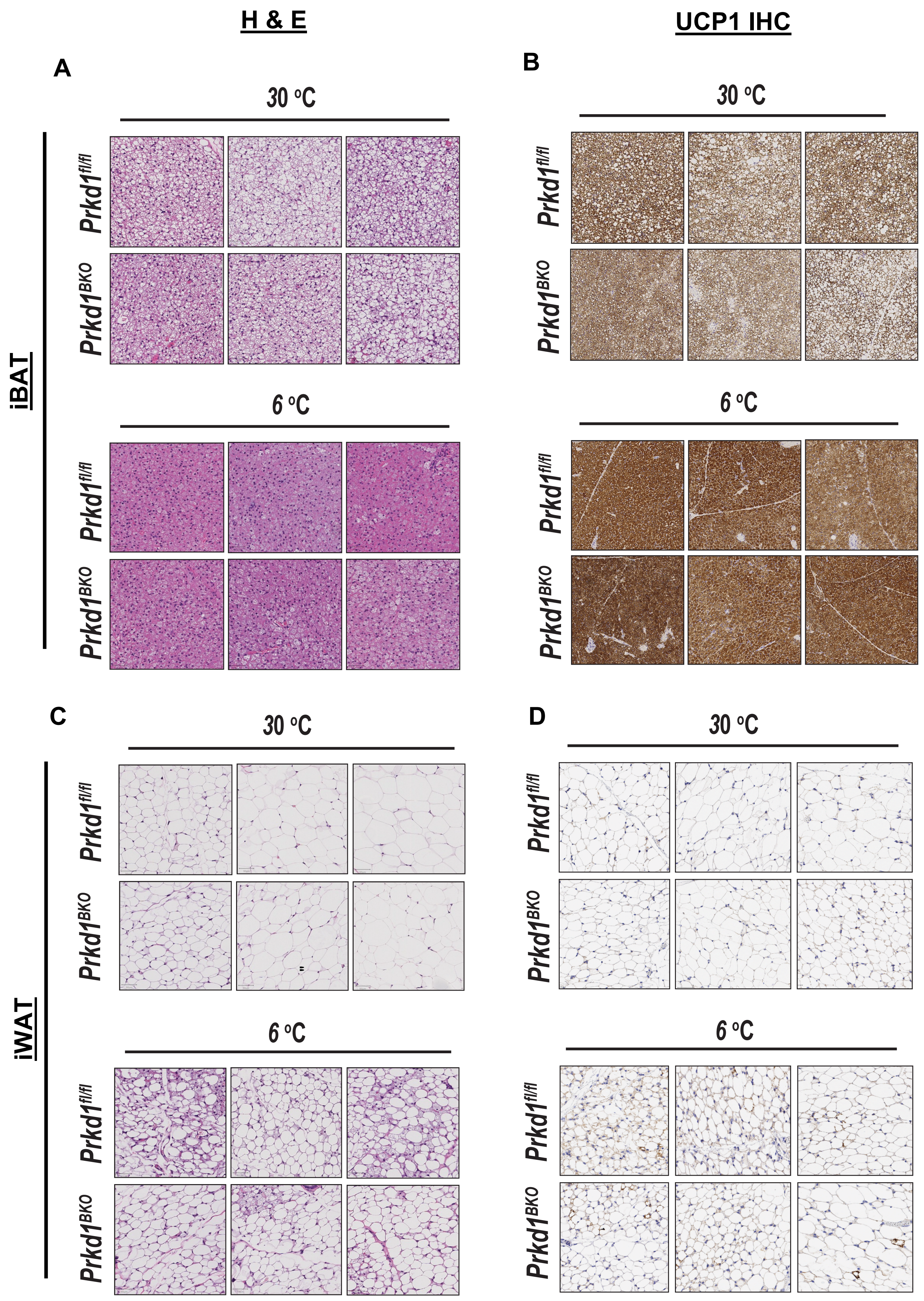

Figure 4

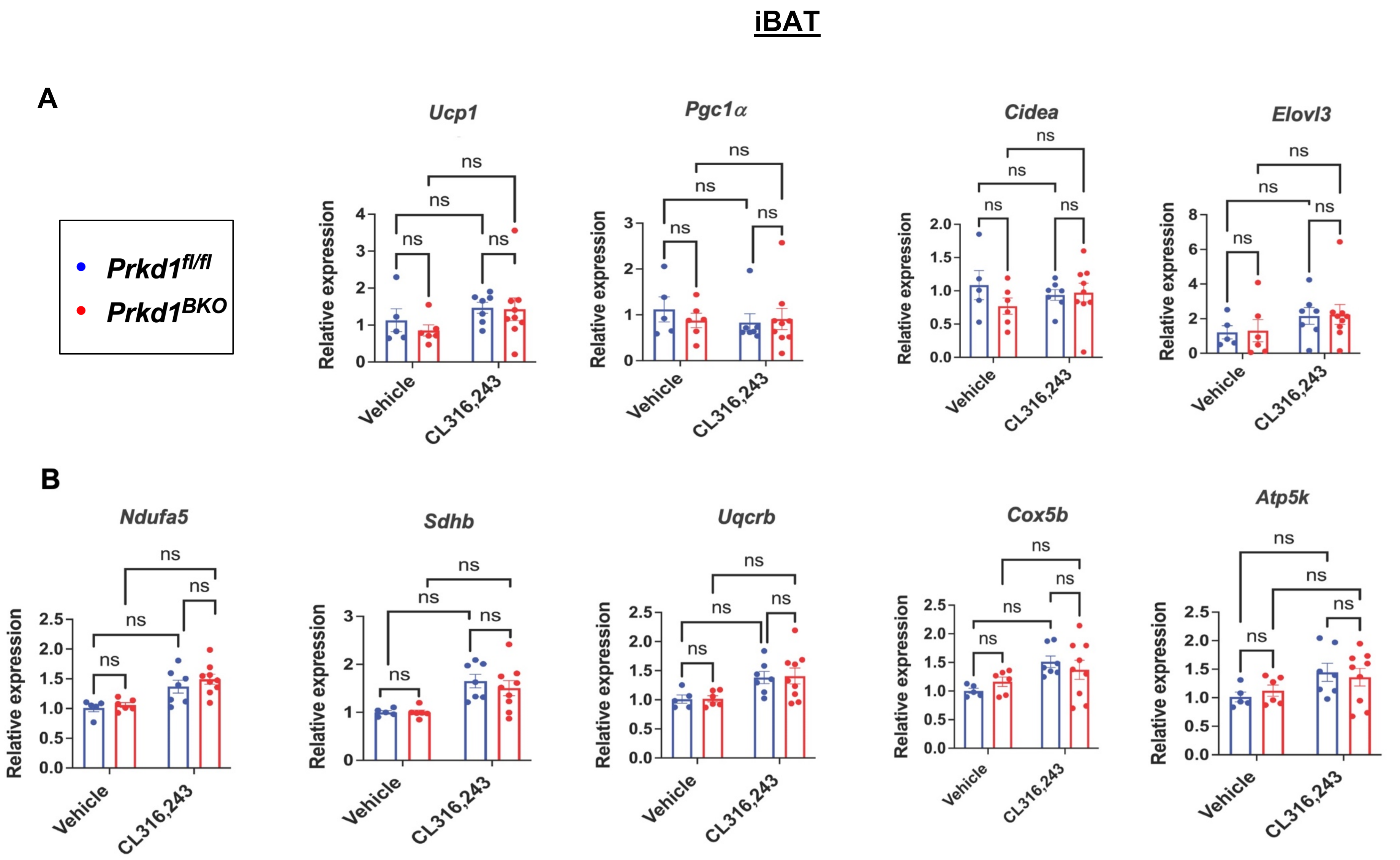
iBAT
A
Prkd1fl/fl
Prkd1BKO
Ucp1
Pgc1α
Cidea
Elovl3
B
Ndufa5
Sdhb
Uqcrb
Cox5b
Atp5k
Relative expression
ns
Vehicle
CL316,243

Figure 5

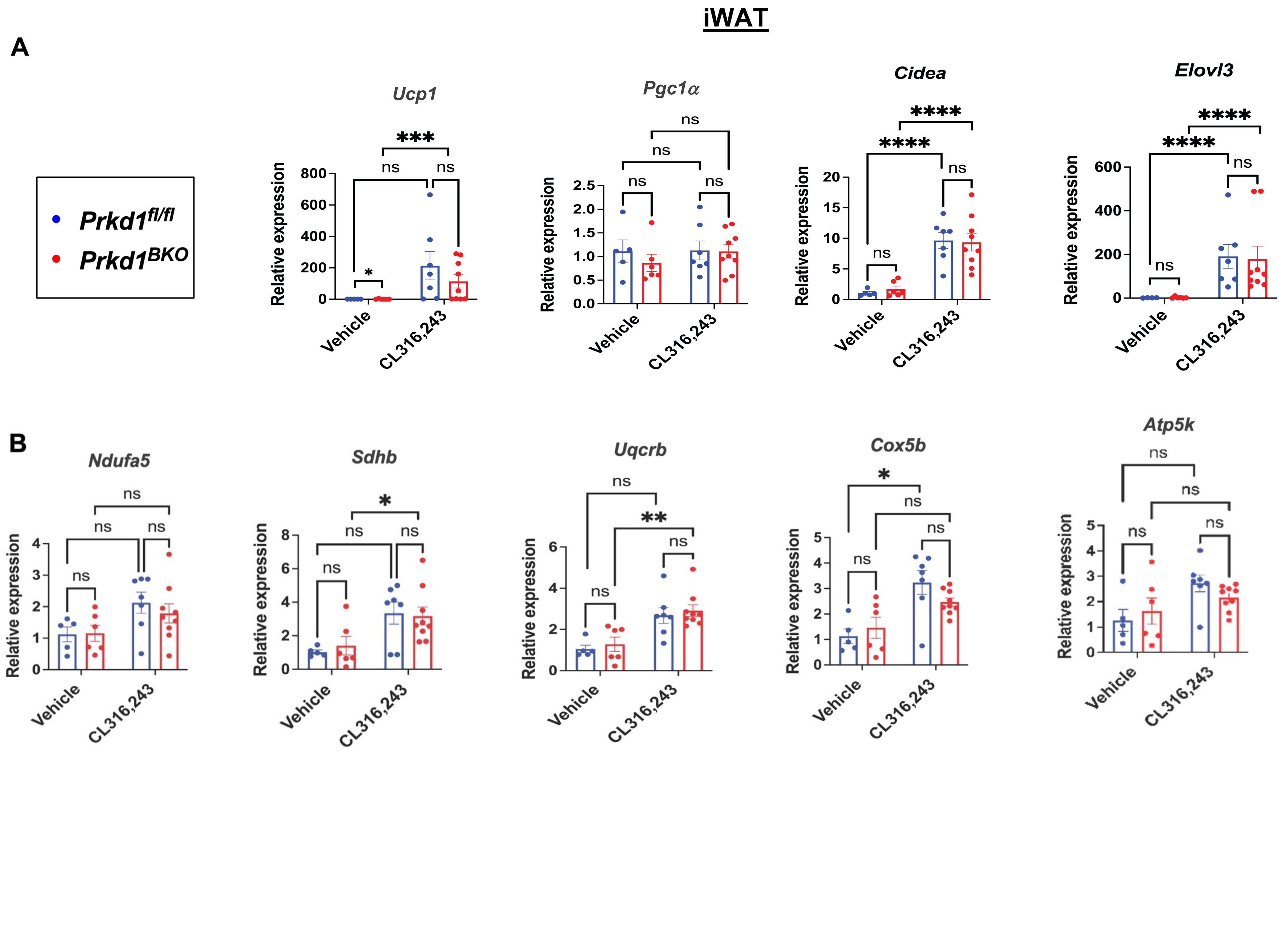

iWAT
A
Prkd1fl/fl
Prkd1BKO
Ucp1
Pgc1α
Cidea
Elovl3
Relative expression
Vehicle
CL316,243
ns
*
***
****
B
Ndufa5
Sdhb
Uqcrb
Cox5b
Atp5k
**

Figure 6

A

B

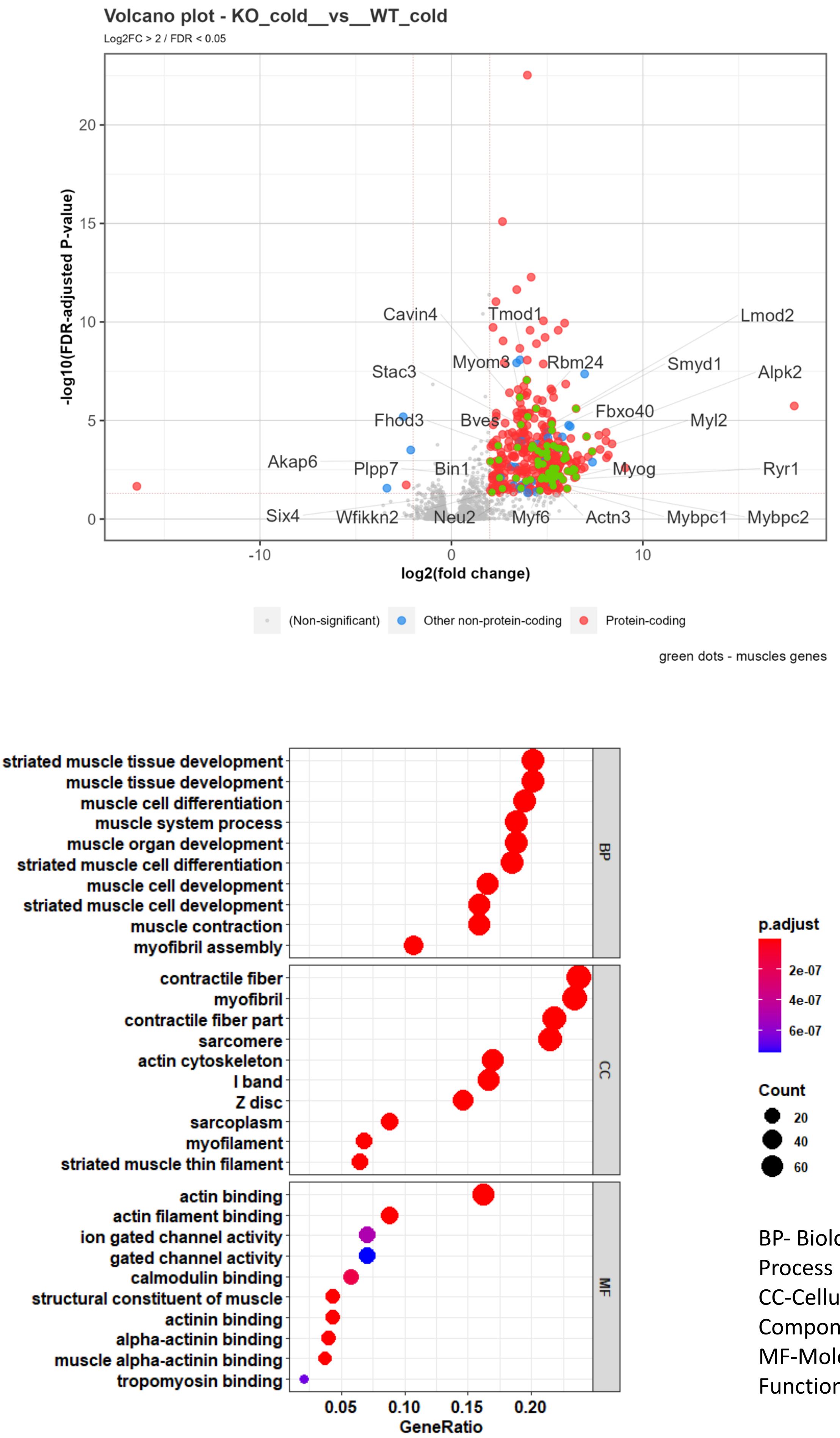

BP- Biological Process
CC-Cellular Component
MF-Molecular Function

Figure 7

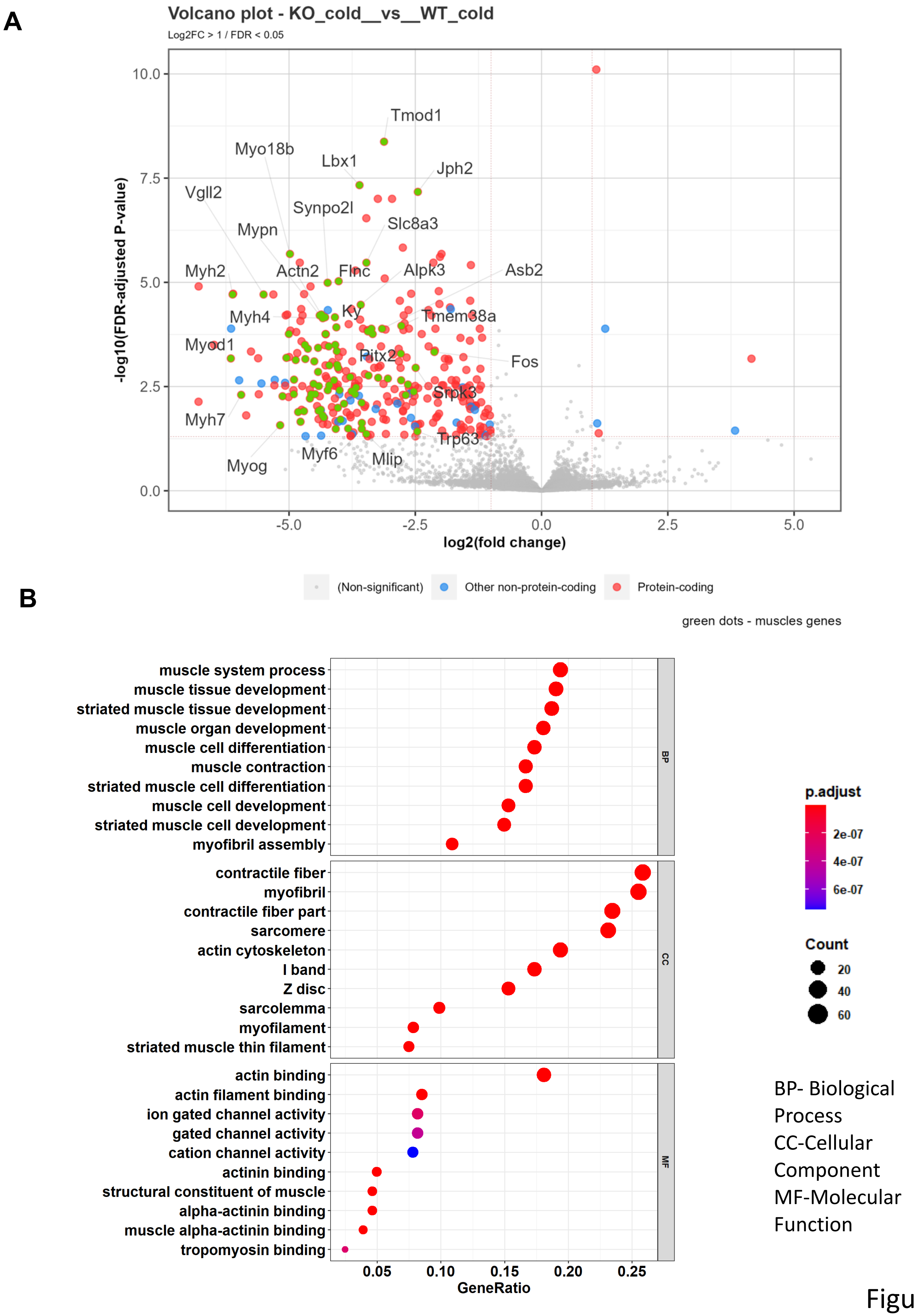
A
Volcano plot - KO_cold__vs__WT_cold
Log2FC > 1 / FDR < 0.05
-log10(FDR-adjusted P-value)
log2(fold change)
10.0
7.5
5.0
2.5
0.0
-5.0
-2.5
0.0
2.5
5.0
Tmod1
Myo18b
Lbx1
Jph2
Vgll2
Synpo2l
Slc8a3
Mypn
Myh2
Actn2
Flnc
Alpk3
Asb2
Ky
Myh4
Tmem38a
Myod1
Pitx2
Fos
Srpk3
Myh7
Trp63
Myf6
Mlip
Myog
(Non-significant)
Other non-protein-coding
Protein-coding
green dots - muscles genes
B
muscle system process
muscle tissue development
striated muscle tissue development
muscle organ development
muscle cell differentiation
muscle contraction
striated muscle cell differentiation
muscle cell development
striated muscle cell development
myofibril assembly
contractile fiber
myofibril
contractile fiber part
sarcomere
actin cytoskeleton
I band
Z disc
sarcolemma
myofilament
striated muscle thin filament
actin binding
actin filament binding
ion gated channel activity
gated channel activity
cation channel activity
actinin binding
structural constituent of muscle
alpha-actinin binding
muscle alpha-actinin binding
tropomyosin binding
BP
CC
MF
0.05
0.10
0.15
0.20
0.25
GeneRatio
p.adjust
2e-07
4e-07
6e-07
Count
20
40
60
BP- Biological Process
CC-Cellular Component
MF-Molecular Function

Figure 8

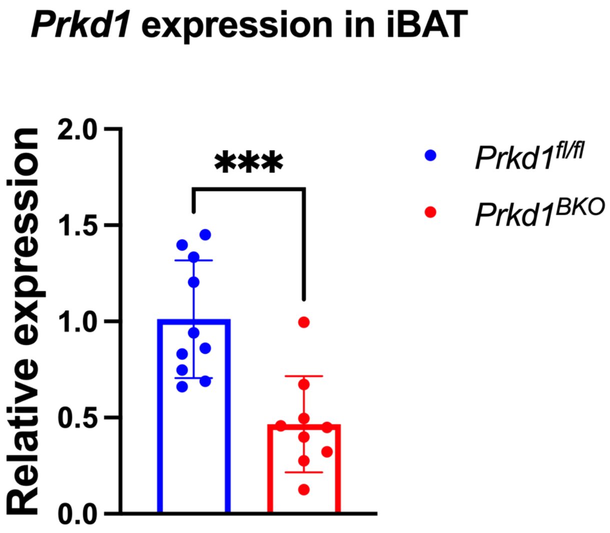

Supplemental Figure 1

8 hour cold RNA-Seq

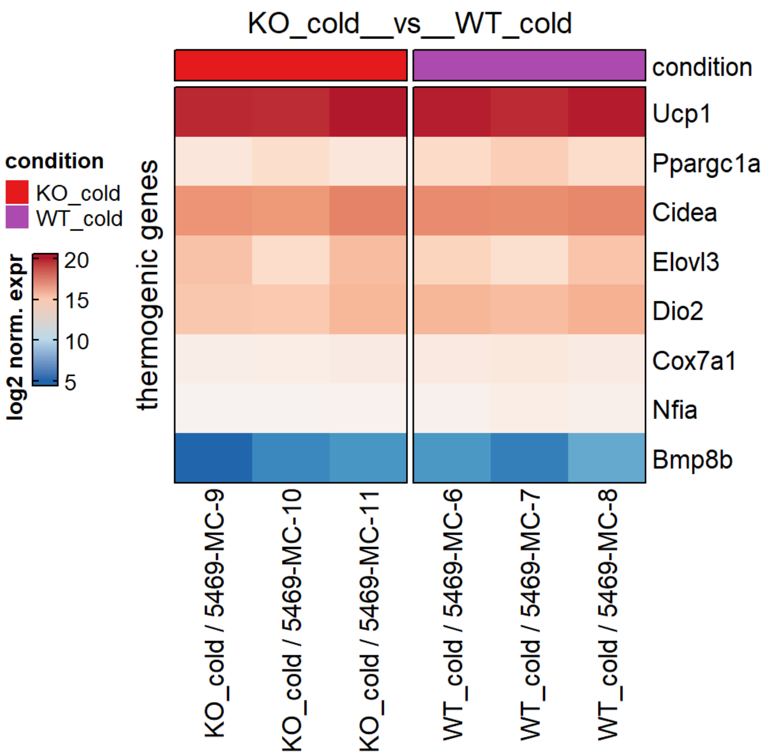

4-day cold RNA-Seq

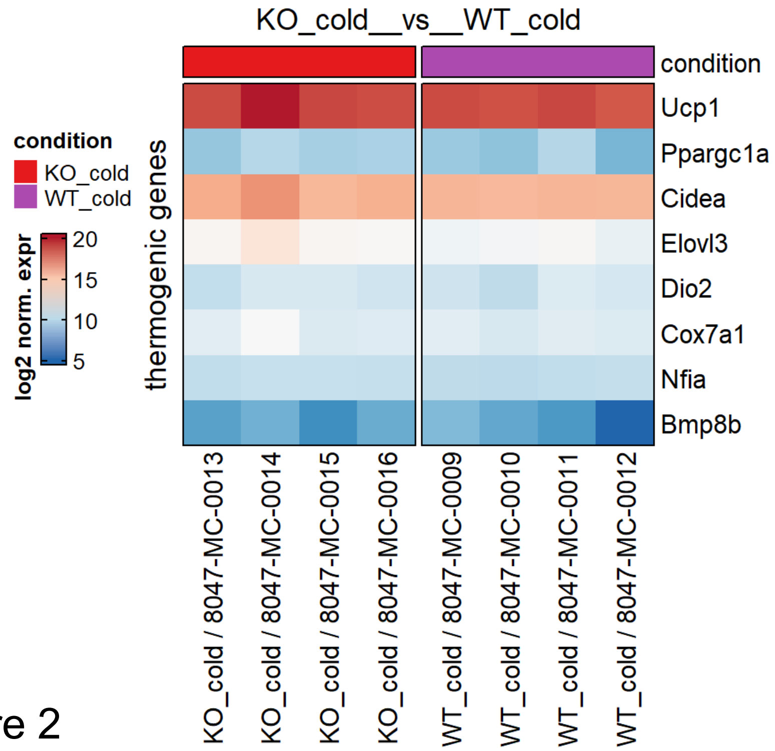

Supplemental Figure 2

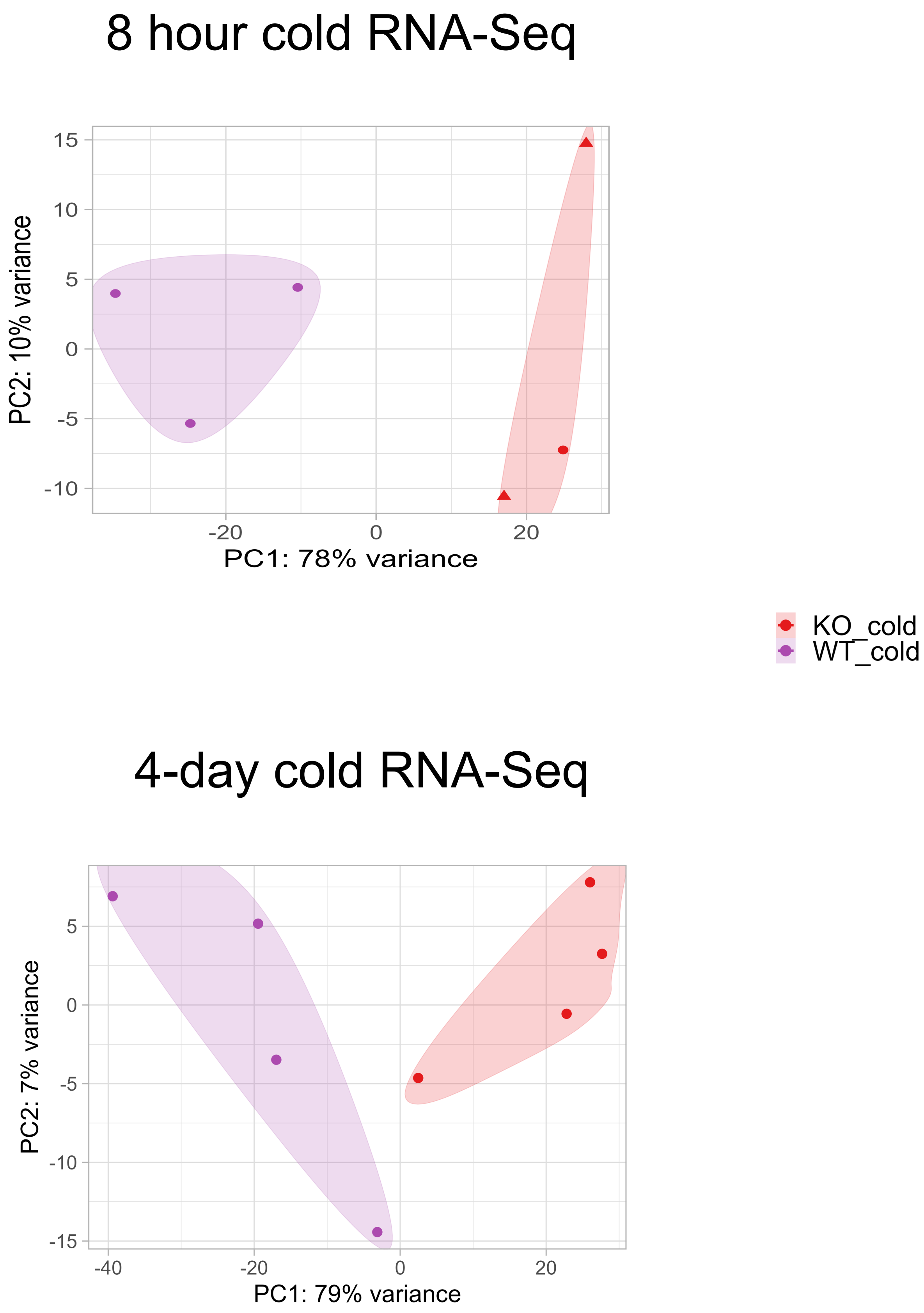

Supplemental Figure 3

8 hour cold RNA-Seq

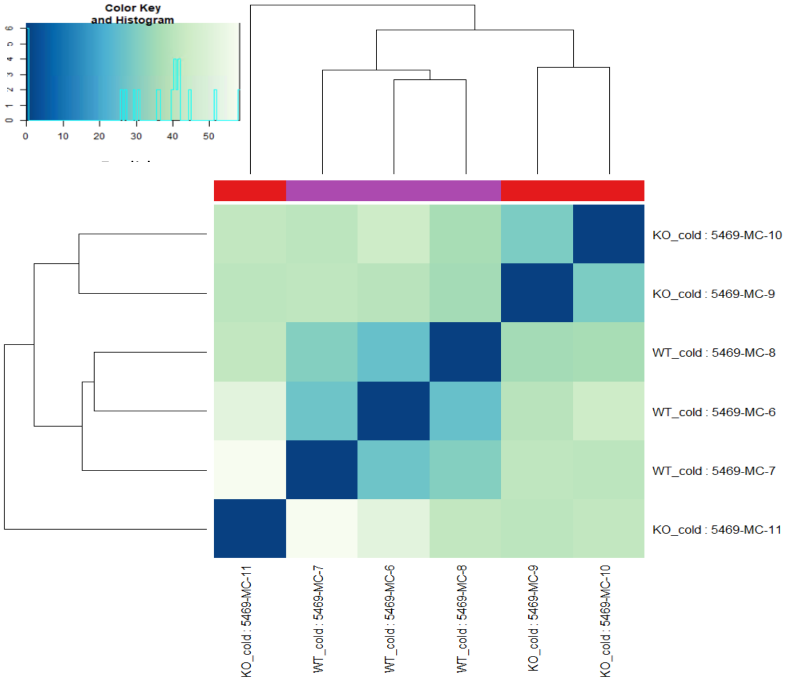

4-day cold RNA-Seq

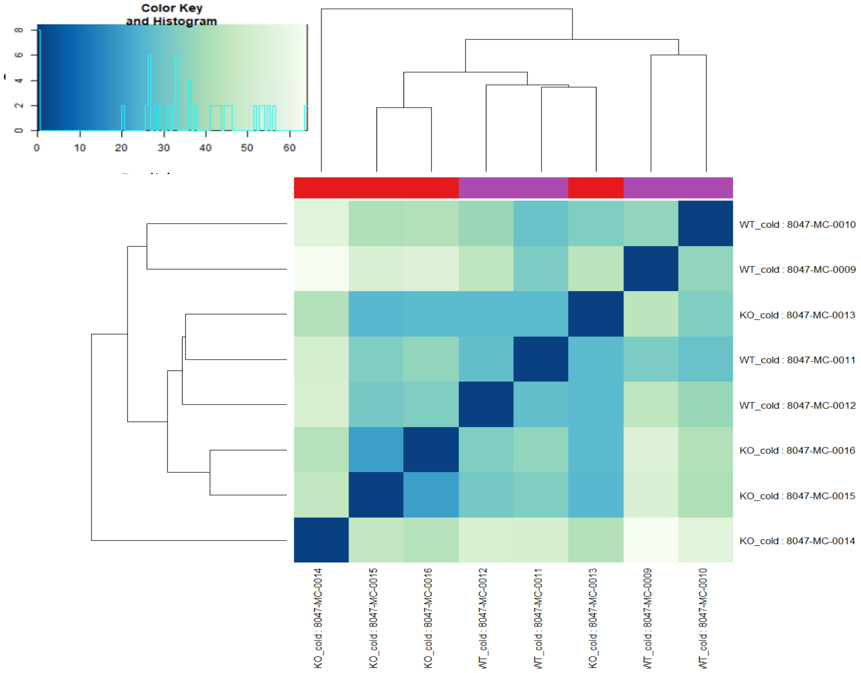

Supplemental Figure 4

Metascape analysis – 8 hour cold RNA-Seq

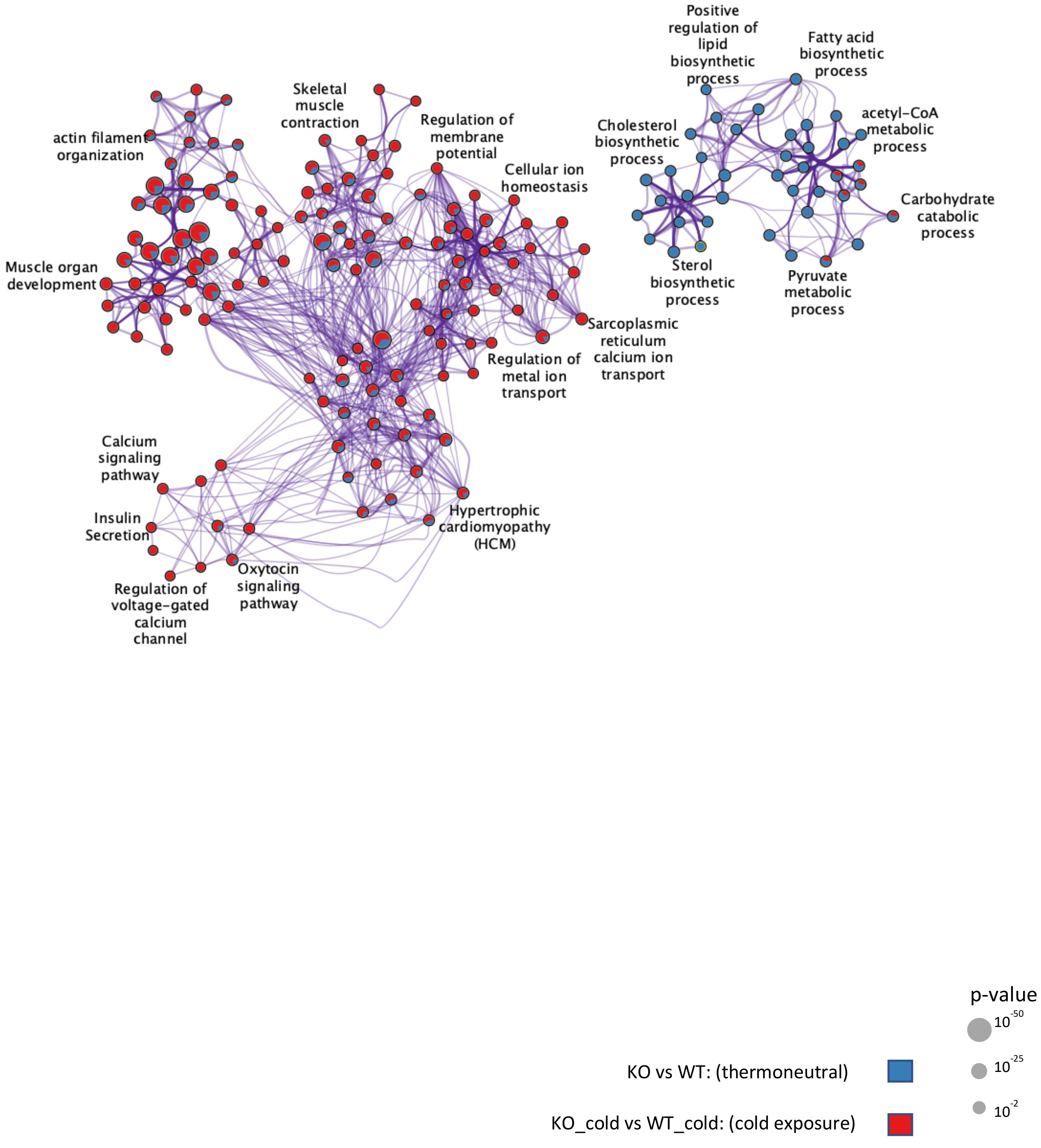

Supplemental Figure 5

Metascape analysis – 4 day cold RNA-Seq

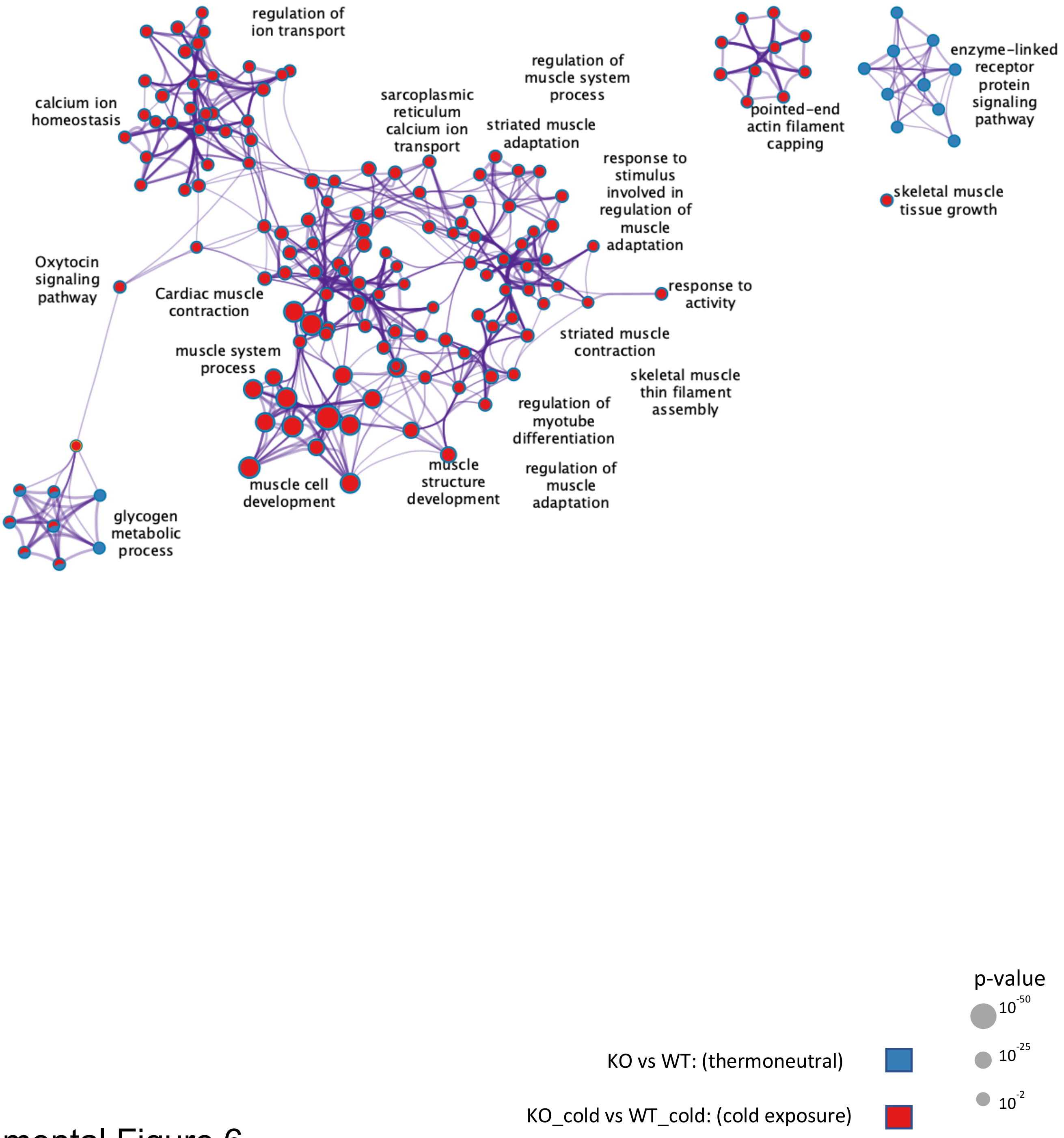

Supplemental Figure 6
